# Microbial functional changes mark irreversible course of Tibetan grassland degradation

**DOI:** 10.1038/s41467-022-30047-7

**Published:** 2022-05-13

**Authors:** Andreas Breidenbach, Per-Marten Schleuss, Shibin Liu, Dominik Schneider, Michaela A. Dippold, Tilman de la Haye, Georg Miehe, Felix Heitkamp, Elke Seeber, Kyle Mason-Jones, Xingliang Xu, Yang Huanming, Jianchu Xu, Tsechoe Dorji, Matthias Gube, Helge Norf, Jutta Meier, Georg Guggenberger, Yakov Kuzyakov, Sandra Spielvogel

**Affiliations:** 1 Department for Crop Sciences, Biogeochemistry of Agroecosystems, University of Goettingen, Buesgenweg 2, 37077 Goettingen, Germany; 2 Department of Geosciences, Geo-Biosphere Interactions, University of Tuebingen, Schnarrenbergstrasse 94-96, 72076 Tuebingen, Germany; 3 Department of Soil Ecology, University of Bayreuth, Dr. Hans-Frisch Strasse 1-3, 95448 Bayreuth, Germany; 4 Institute of Ecological Environment, Chengdu University of Technology, 610059 Chengdu, China; 5 Institute of Microbiology and Genetics and Goettingen Genomics Laboratory, University of Goettingen, Grisebachstrasse. 8, 37077 Goettingen, Germany; 6 Department of Soil Science, University of Kiel, Hermann-Rodewald-Strasse 2, 24118 Kiel, Germany; 7 Faculty of Geography, University of Marburg, Deutschhausstrasse 10, 35032 Marburg, Germany; 8 Environmental Control, Northwest German Forest Research Institute, Graetzelstrasse 2, 37079 Goettingen, Germany; 9 Department of Botany, Senckenberg Museum of Natural History Goerlitz, 02806 Goerlitz, Germany; 10 Netherlands Institute of Ecology, Department of Terrestrial Ecology, Postbus 50, 6700 AB Wageningen, the Netherlands; 11 Key Laboratory Ecosystem Network Observation and Modeling, Institute of Geographic Science and Natural Resources Research, Chinese Academy of Science, 11A Datun Road, 100101 Beijing, China; 12 CAS Center for Excellence in Tibetan Plateau Earth Sciences, Chinese Academy of Sciences (CAS), 100101 Beijing, China; 13 Beijing Genomics Institute, BGI Park No. 21 Hongan 3rd Street, Yantian District, 518083 Shenzhen, China; 14 Center for Mountain Futures, Kunming Institute of Botany, Chinese Academy of Sciences, 650201 Kunming, China; 15 Institute of Tibetan Plateau Research, Chinese Academy of Sciences, No. 16 Lincui Road, Chaoyang District, 100101 Beijing, China; 16 Soil Science of Temperate Ecosystems, University of Goettingen, Buesgenweg 2, 37077 Goettingen, Germany; 17 Department of River Ecology, Department of Aquatic Ecosystems Analysis and Management, Helmholtz Centre for Environmental Research GmbH UFZ, Brueckstrasse 3a, 39114 Magdeburg, Germany; 18 Institute for Integrated Natural Sciences, University of Koblenz-Landau, Universitätsstrasse. 1, 56070 Koblenz, Germany; 19 Institute of Soil Science, Leibniz Universität Hannover, Herrenhäuser Strasse 2, 30419 Hannover, Germany; 20 Agricultural Soil Science, University of Goettingen, Buesgenweg 2, 37077 Goettingen, Germany

**Keywords:** Carbon cycle, Carbon cycle

## Abstract

The Tibetan Plateau’s *Kobresia* pastures store 2.5% of the world’s soil organic carbon (SOC). Climate change and overgrazing render their topsoils vulnerable to degradation, with SOC stocks declining by 42% and nitrogen (N) by 33% at severely degraded sites. We resolved these losses into erosion accounting for two-thirds, and decreased carbon (C) input and increased SOC mineralization accounting for the other third, and confirmed these results by comparison with a meta-analysis of 594 observations. The microbial community responded to the degradation through altered taxonomic composition and enzymatic activities. Hydrolytic enzyme activities were reduced, while degradation of the remaining recalcitrant soil organic matter by oxidative enzymes was accelerated, demonstrating a severe shift in microbial functioning. This may irreversibly alter the world´s largest alpine pastoral ecosystem by diminishing its C sink function and nutrient cycling dynamics, negatively impacting local food security, regional water quality and climate.

## Abstract (web summary)

The Tibetan Kobresia pastures store 2.5% of the world’s soil organic carbon. Here the authors show that soil degradation and microbial shifts may irreversibly diminish the carbon sink function and accelerate nutrient losses.

## Introduction

The Tibetan Plateau (TP) hosts the world’s largest high-altitude grasslands, contributing 2.5% to global SOC stocks^1^ but covering only 0.3% of the Earth’s total terrestrial area. It influences the Asian monsoon climate^2^, is the water source for one-fifth of the global population^3^ and provides grazing grounds for >8 million sheep, yaks, and goats^4^. Approximately one-fifth of the TP is covered by *Kobresia* grasslands^2^. *Kobresia pygmaea* forms a 2–4 cm high grazing lawn with low shoot biomass but very compact root mats (root-to-shoot ratio >20)^5^, induced by a long history of low-to-moderate grazing intensity, which increases belowground carbon allocation and root biomass^6,7^. *Kobresia pygmaea*’s dense root network protects it from trampling-induced soil erosion and enables fast regrowth after defoliation^8^.

Pasture degradation has increased dramatically in recent decades^2,3^, and about 30% of the Tibetan grasslands are considered to be degraded^9^. This has indisputably severe consequences for ecosystem functions, most importantly the major decline in SOC and N storage. Three mechanisms contribute to this—erosion, decreased C and N input, and increased soil organic matter (SOM) mineralization—but their relative importance remains unclear^10^. We conducted a meta-analysis including 594 single observations from 49 literature studies published between 2002 and 2020 (Supplementary Table 1) to quantify the SOC and N losses for *Kobresia pygmaea*’s core area. In a detailed field study, we determined the relative contributions of erosion and net mineralization to SOC losses and identified the underlying changes in microbial community structure and functioning. For this, we categorized six successive stages of degradation, from intact *Kobresia* root mats (S0) to stages with increasing extents of surface cracks (S1–S4) to bare soil patches without root mats (S5) (Supplementary Fig. 1).

We hypothesize that substantial parts of the *Kobresia* pastures are close to a critical point of microbial functioning changes with substantial consequences for SOC and N storage. We further hypothesize that these abrupt shifts are characterized by changes in SOM quality and quantity that are coupled by feedback loops to microbial community structure and functions, which in turn control C and N mineralization.

## Results

### Root-mat cracking and soil erosion

**Fig. 1:**
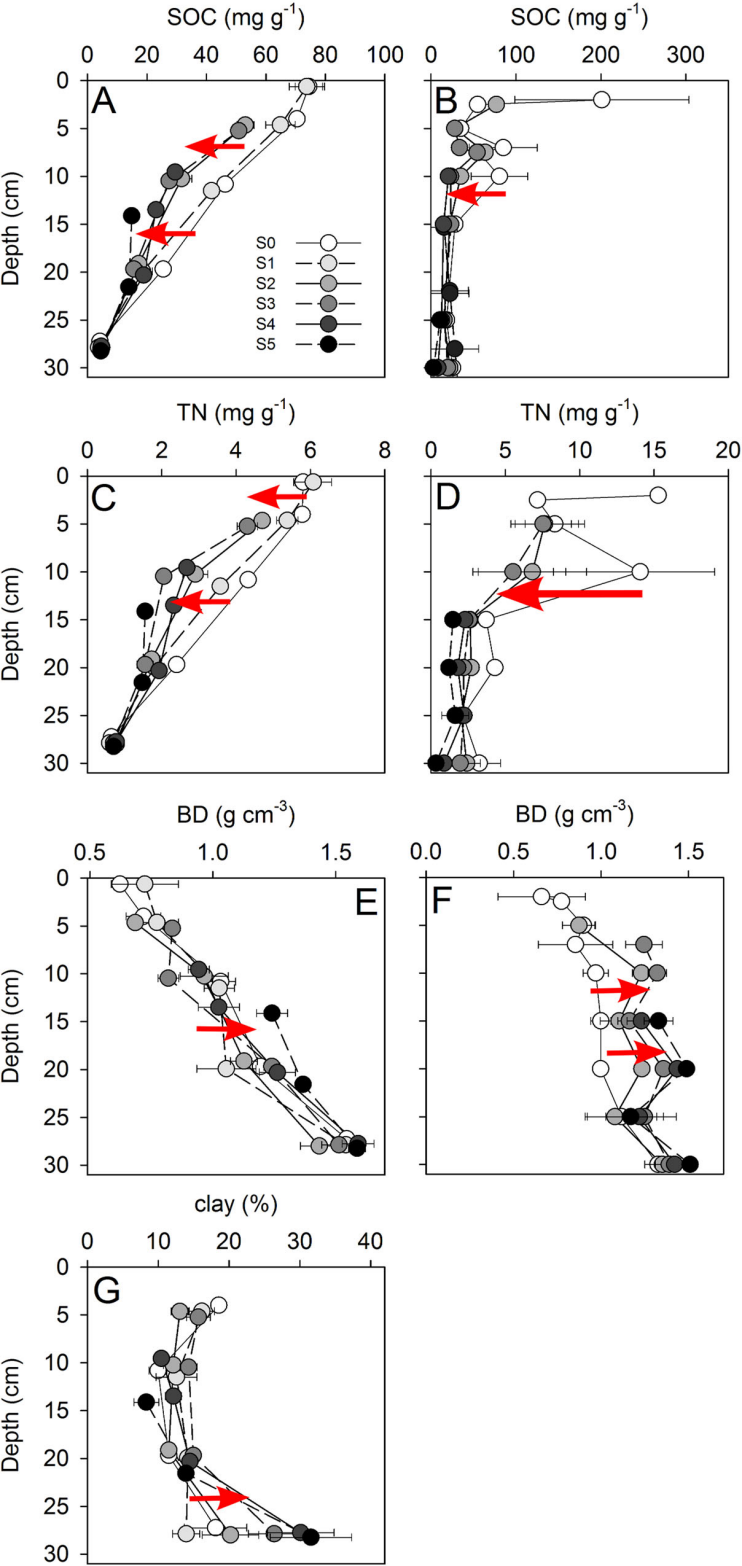
Changes in soil characteristics with depth according to degradation stage. **A** soil organic carbon (SOC) core study site, **B** SOC literature study, **C** total nitrogen (N) core study site, **D** total N literature study, **E** bulk density (BD) core study site, **F** BD literature study, **G** clay content core study site. All parameters are presented for each soil horizon at the midpoint of the depth increment. Error bars display standard error. Exact data are provided in Supplementary Table 2.

**Fig. 2:**
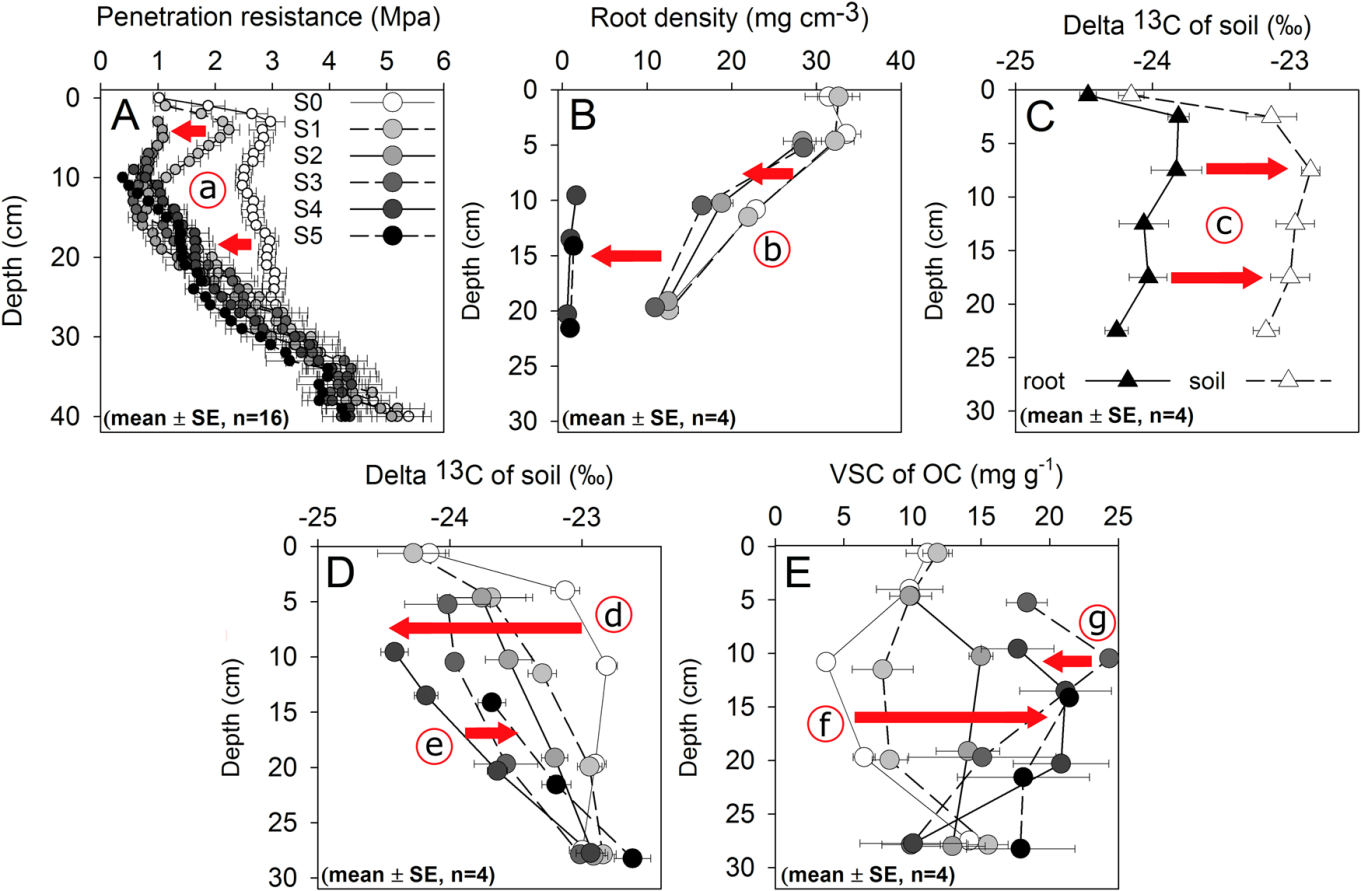
Changes in soil characteristics with depth according to degradation stage. **A** Penetration resistance, **B** root density, **C** δ^13^C of soil and roots, **D** δ^13^C of soil organic carbon (SOC), **E** content of lignin monomers vanillyl, syringyl, and cinnamyl (VSC). All parameters (means ± SE, *n* = 4) are presented for each soil horizon at depth midpoint, except for penetration resistance (**A**) and δ^13^C values of soil and roots (**C**), which are shown in 1 and 5 cm increments, respectively. Exact data are given in Supplementary Table 2. Progressive changes along the degradation sequence can be explained by the following processes: (a) root-mat cracking by desiccation and frost, (b) root death and decomposition, (c) kinetic ^13^C fractionation during root decomposition, (d) SOC loss due to reduced root carbon (**C**) input and greater SOC mineralization, (e) relative lignin accumulation and ^13^C_SOC_ depletion (S0–S3), (f) relative lignin accumulation during stages S0–S3, (g) lignin degradation and ^13^C_SOC_ enrichment (S4, S5).

The formation of polygonal surface cracks is widespread between the Qilian Mountains and the Himalayas (Supplementary Fig. 1). These surface cracks represent the early stages of a degradation process that, in its final stage (S5), is associated with a loss of 42% of SOC compared to non-degraded pastures (Fig. 1A, B; Supplementary Fig. 2). Nitrogen losses were comparably high (33%; Fig. 1C, D; Supplementary Fig. 2). Soil erosion induced preferential loss of the easily erodible fine particles, resulting in a relative accumulation of coarser soil material from S0 to S5 (Fig. 1G, Supplementary Fig. 3), with soil clay content being 60% lower at the most degraded sites compared to intact pastures (Fig. 1G; Supplementary Fig. 3). At the most extreme degradation stage (S5) of our own study site, 81 kg m^−2^ of the most fertile SOC- and N-rich topsoil had been lost to erosion. This corresponds to 5 kg C m^−2^ or 45% of the total soil C stock (Fig. 1A), in agreement with the mean soil C loss found in the literature study (42%). Degradation of the *Kobresia* turf was furthermore associated with a large decrease in penetration resistance (Fig. 2A) and root density (Fig. 2B) from S1 to S5. Intensified degradation decreased vegetation cover, leaving the soil prone to erosion with extended size and depth of cracks (Supplementary Table 2).

### SOC losses through mineralization and decreased root carbon input

The SOC and N contents were closely positively correlated with root density (Supplementary Fig. 4), and showed a consistent relationship in isotopic composition (δ^13^C) with the associated roots at each depth (Fig. 2C). This demonstrates the importance of roots as the dominant SOC source for intact *Kobresia* pastures. An association of increasing δ^13^C values with declining SOC content (Fig. 2D) due to kinetic isotope fractionation during decomposition was observed only at the final degradation stage. With degradation from S1 to S4, the SOC content in the upper 20 cm decreased (Fig. 1A, B), while δ^13^C values already decreased from S0 to S1 (Fig. 2D). We quantified the sum of lignin monomers vanillyl, syringyl, and cinnamyl as an indicator of complex organic compounds^11^. Vanillyl, syringyl, and cinnamyl contents increased from S0 to S4 (Fig. 2E), indicating selective lignin preservation during early degradation stages. The close negative correlation between δ^13^C and vanillyl, syringyl, and cinnamyl contents in the mineral horizon indicates the selective enrichment of the isotopically light lignin (Supplementary Fig. 5A). However, the decrease of vanillyl, syringyl, and cinnamyl contents from S4 to S5 in the mineral soil (Fig. 2E), accompanied by ^13^C enrichment (Fig. 2D) and a decrease in SOC content (Supplementary Fig. 5B), indicates a pronounced lignin decomposition at the final degradation stage (S5).

### Soil microbial community structure and functions

The bacterial (Supplementary Figs. 6A, and 9) and fungal (Supplementary Figs. 6B, and 10) community composition changed significantly (*p* < 0.05, MANOVA) along the degradation sequence. Bacterial groups (Supplementary Table 3) associated with decomposition of low-molecular-weight organic compounds, e.g., *Actinobacteria* (Supplementary Figs. 7A, and 9), declined with pasture degradation, whereas lignin-degrading groups such as *Rhizobiales* increased (Supplementary Fig. 7D). Nitrifying (*Nitrospirales* and *Nitrosomonadaceae*; Supplementary Fig. 7B, C) and denitrifying (*Pseudomonadales*; Supplementary Fig. 7E) bacteria increased from S0 to S3 and then declined towards S5. These shifts indicate severe changes in N-cycling towards the accelerated microbial transformation of organic N to mineral N in the early stages of degradation. Similarly, fungal groups specialized in efficient lignin degradation increased in relative abundance at later degradation stages, as evidenced by *Agaricomycetes*^12,13^ (Supplementary Fig. 7F), which include the brown-rot and white-rot fungi. Correspondingly, groups generally incapable of efficient lignin degradation^12^, such as *Ascomycota* (Supplementary Fig. 7G), decreased along the degradation sequence (Supplementary Fig. 10a, b). After a peak at degradation stage S2, *Kobresia pygmaea* successively lost its arbuscular mycorrhizal fungal partners (*Glomeromycota*, Supplementary Fig. 7H), which were replaced by ectomycorrhizal ones (*Thelephoraceae* and *Inocybaceae*, Supplementary Fig. 7I). When *Kobresia pygmaea* largely disappeared (S5), the abundance of its ectomycorrhizal partners also declined and the first arbuscular mycorrhizal partners of the newly establishing pioneer plants appeared (Supplementary Fig. 7H).

**Fig. 3:**
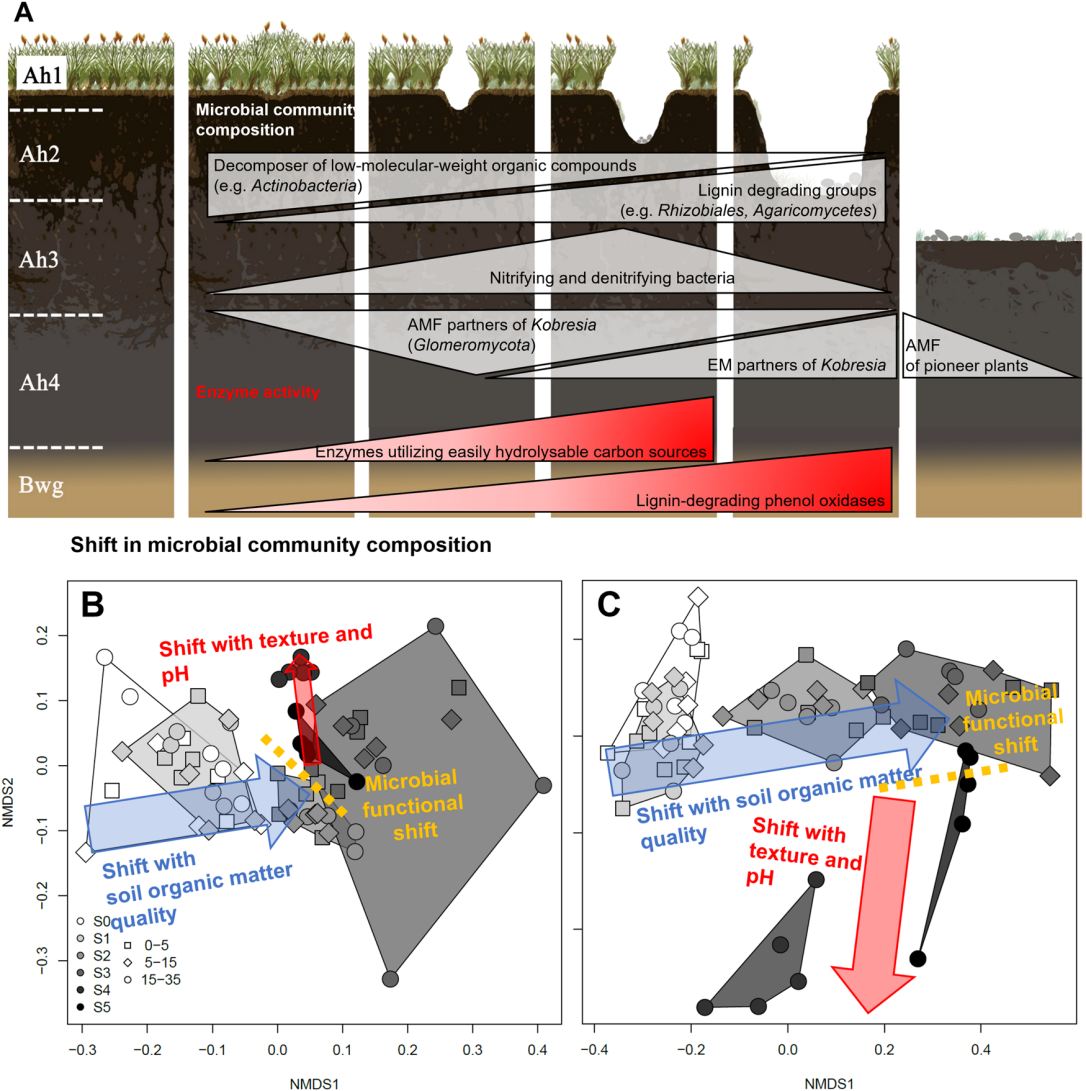
Overview of generalized changes in microbial community composition and functioning along the degradation sequence from intact (S0) to severely degraded (S5) stage. Generalized changes in microbial community composition and enzyme activities (**A**). Simplified non-metric multidimensional scaling (NMDS) plots derived from terminal restriction fragment length polymorphism (t-RFLP) data for the bacterial (**B**) and fungal (**C**) communities. Shaded areas mark each degradation stage, symbols indicate soil depth, and arrows show most important driving factors (canonical correspondence analysis, *p* < 0.05). Individual variables underlying the processes described by the blue and red arrows are shown in Supplementary Fig. 6.

Non-metric multidimensional scaling (NMDS), including C/N, δ^13^C, total N, δ^15^N, total phosphorus (P), pH, cation exchange capacity, calcium, potassium, iron and aluminum content, clay content and vanillyl, syringyl, and cinnamyl content as explanatory factors revealed a strong correlation between microbial community structure and SOC quality (C/N, δ^13^C, N, P and vanillyl, syringyl, and cinnamyl) during early degradation stages (Fig. 3). However, changes in microbial community structure during later degradation stages (from S3 to S5) were mainly driven by abiotic soil properties such as increasing pH and decreasing clay content, which had a stronger effect on fungi than on bacterial communities.

Maximal activities of enzymes for utilizing easily hydrolyzable C sources (β-glucosidase and xylanase) significantly increased from S0 to S3 and declined when pasture degradation reached stage S4 (Supplementary Figs. 7J, and 12). Likewise, the activity of N- and P-mobilizing enzymes (urease and alkaline phosphatase) declined from S3 to S4 (Supplementary Figs. 7J, and 12). In contrast, the activity of lignin-degrading phenol oxidases increased steadily with the degradation from S2 to S4 along with the relative increase in vanillyl, syringyl, and cinnamyl monomers (Supplementary Figs. 7J, and 12).

### Disentangling processes contributing to SOC losses

**Fig. 4:**
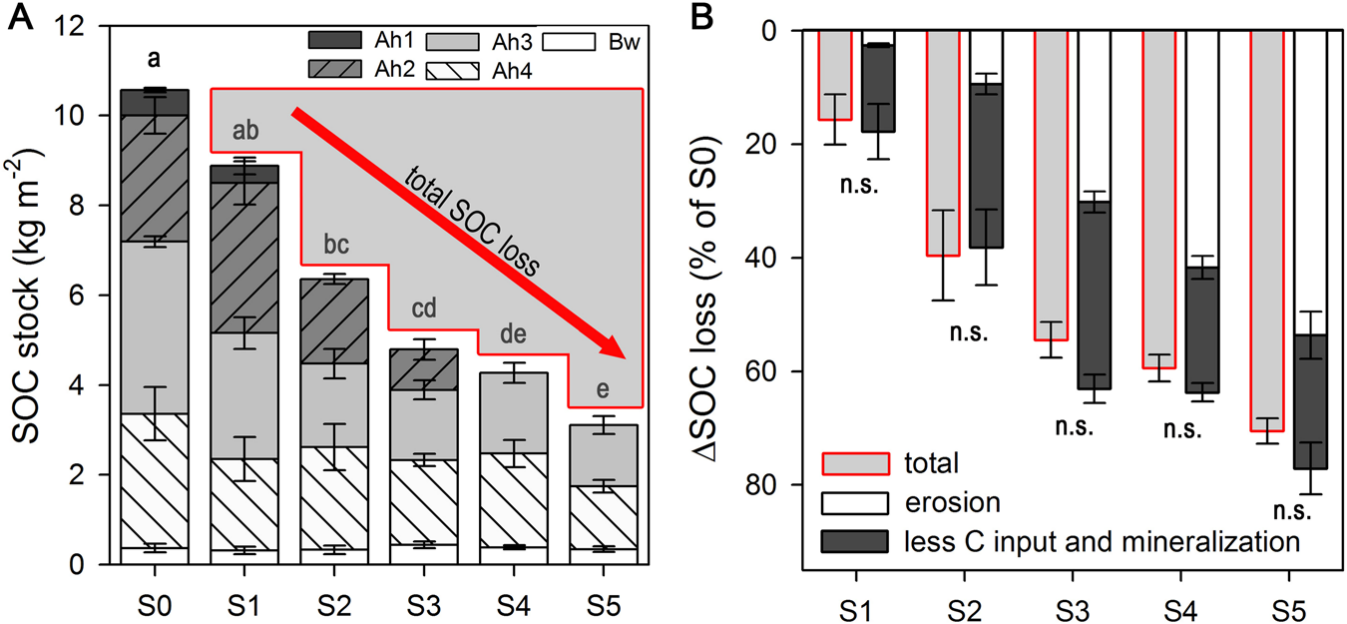
Soil organic carbon (SOC) stocks and losses with increasing degradation. Total SOC stocks along the degradation sequence (S0–S5) down to 30 cm for each horizon (**A**). SOC losses by erosion and by reduced C input and increased SOC mineralization (**B**) during degradation (S1–S5). Lowercase letters indicate significant differences in SOC stocks between degradation stages. The absence of significant differences (n.s.) between quantified total SOC losses (red outline) and the calculated sum of erosion- and mineralization-induced SOC losses confirms the validity of the estimations. Error bars display standard error.

In the study area, SOC stocks at 0–30 cm depth declined strongly by 7.5 kg C m^−2^ from the stage of intact *Kobresia* root mats (S0) to that of bare soil patches (S5). This corresponds to a 45% reduction (Fig. 4) and matches well with the mean SOC loss observed for degraded areas across the whole TP (42%). SOC losses were attributed to topsoil erosion, which accounted for two-thirds of the decline (for S5: 5.0 kg C m^−2^, Fig. 4B), with the combined effects of decreasing root carbon input and accelerated mineralization of root litter and SOC accounting for the remaining one-third of the decline (2.5 kg C m^−2^; Fig. 4B).

## Discussion

**Fig. 5:**
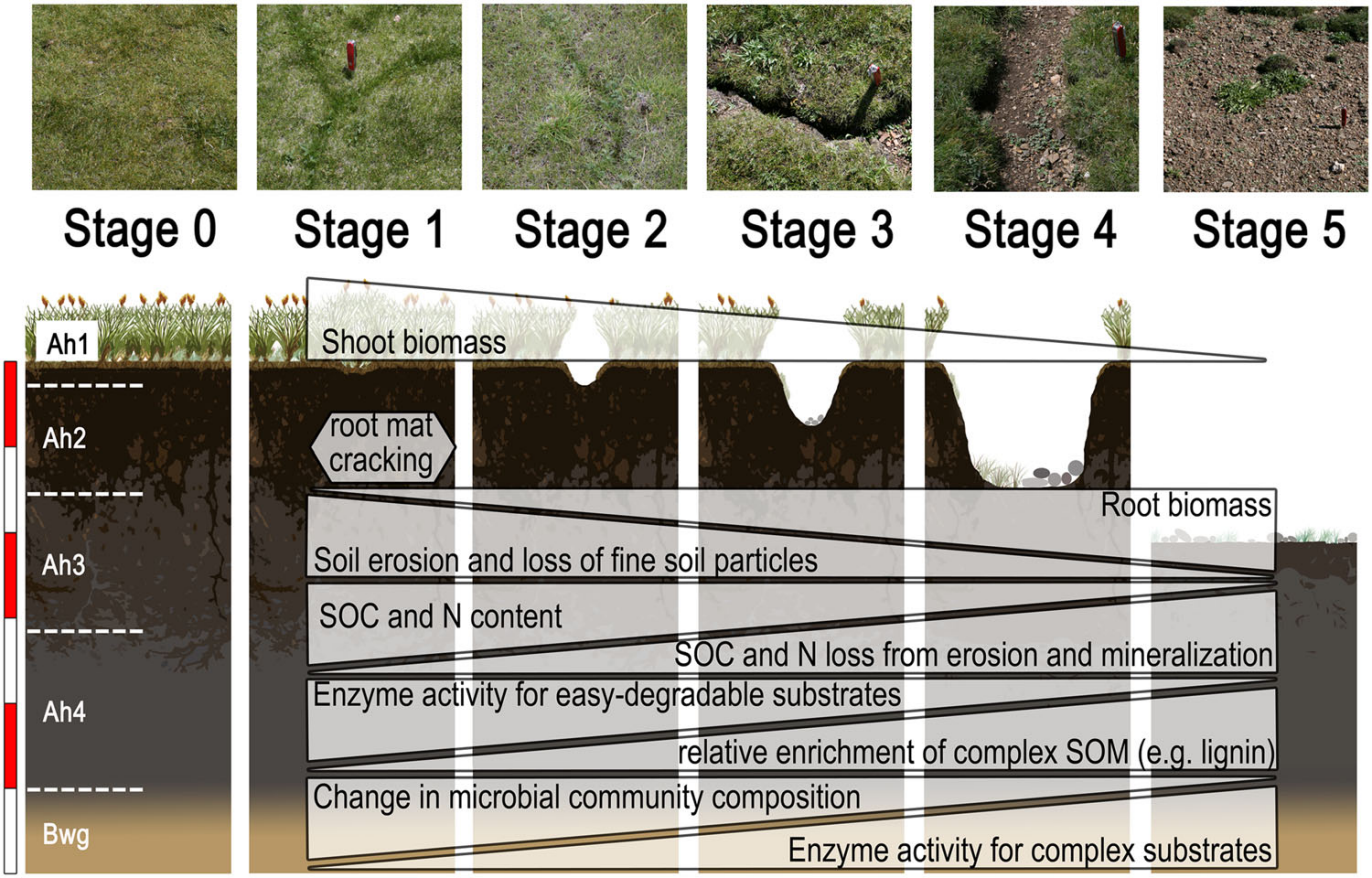
Overview of the degradation sequence from the intact (S0) to severely degraded (S5) stage, showing the driving forces of degradation. Polygons describe main biotic and abiotic degradation mechanisms with a focus on soil organic carbon (SOC) pools, in accordance with the hypotheses. The white/red scale on the left shows a soil depth of 30 cm in 5 cm increments.

Above- and belowground linkages of organic matter and energy flux play a central role in the responses of the TP *Kobresia* ecosystem to anthropogenic forcing and climate warming, as reported for mountain ecosystems worldwide^14^. Severe soil degradation of the *Kobresia* ecosystem results from complex reinforcing interactions of biotic and abiotic processes, as well as anthropogenic pressure (Fig. 5). Frost events at the beginning of the cold season, when the soil is moist, cause the formation of polygonal surface cracks, a process severely enhanced by permafrost thaw^15^. Overgrazing-induced trampling, followed by root death and decomposition, expand these surface cracks (S1, S2). Exacerbated polygonal cracking then promotes soil erosion with preferential loss of fine-textured material, containing SOC and nutrients (S3–S4). This implies, among others, a strong reduction in the water holding capacity with as yet unforeseeable consequences for the water supply and quality of one-fifth of the world’s population. Moreover, erosion of soil material and the associated loss of SOC carry severe consequences for ecosystem energy, water, and C fluxes, e.g., regionally earlier initiation of convection and cloud generation^2^, affecting the strength and variability of the TP summer monsoon^2^.

δ^13^C patterns in roots and SOC in undegraded pastures demonstrate that SOC accumulation depends heavily on root biomass input and rhizodeposition. A close correlation between SOC stocks and root density across all degradation stages demonstrates that decreasing C input with degradation is a major driver of decreasing SOC content. Decreasing SOC content along the degradation gradient indicates that root necromass was not stabilized in degraded soils, and consequently labile constituents of root necromass were rapidly mineralized. The large root litter input from dying *Kobresia* stimulated microorganisms to decompose the old SOC pool (root litter priming). This was confirmed by the increased activity of hydrolyzing enzymes during the early degradation stages. Such priming effects were triggered by the three to four times higher C/N ratio in roots compared to soil, driving microbes in this N-limited ecosystem^5^ to decompose SOM to obtain N (N mining)^16^. Similar effects of N limitation were observed along a thawing permafrost chronosequence in grassland soils of the TP, where N limitation in the late stages of permafrost collapse induced SOC decomposition through priming effects^17^. In the case of permafrost thawing, a decrease in metabolic efficiency was the major mechanism underlying priming, without a response in hydrolytic enzyme activity. The root litter decomposition observed along our degradation sequence was, however, clearly related to an increase in hydrolytic enzyme activity from S0 to S3. Thus, the successive degradation and subsequent mineralization of the organic N stocks increased the abundance of nitrifying and denitrifying bacteria during the early degradation stages (S0–S3). Using N-cycling gene abundances (*amoA*), Che et al.^18^ identified an up to five-fold increase in nitrifiers associated with a major shift in the N cycle during the degradation of Tibetan pasture meadows. Nitrification produces easily leachable nitrate, a limiting substrate for denitrification in alpine meadows^19^, which consequently increases in periods of high soil moisture or in moist soil microhabitats. We observed an increase in N-hydrolyzing enzymes, an increase in plant litter decomposers, and an increasing abundance of nitrifier- and denitrifier-hosting bacterial orders and families (*Nitrospirales* and *Nitrosomonadaceae* (Supplementary Fig. 7B, C) and *Pseudomonadales* (Supplementary Fig. 7E), respectively) from degradation state S0–S3. This suggests that all three processes - N mineralization, nitrification, and denitrification—increase along the degradation gradient, which leads to severe nitrogen losses from this ecosystem via leaching, emissions of nitrous oxide and nitrogen gas, and NH_3_ volatilization^20^. Quantification of the net and gross rates of these processes^21^ as well as corresponding gene abundances^22^ confirmed that these three key steps of the N cycle accelerate with increased grazing intensity in Tibetan meadows. Concomitant increases in mineral N availability^21^ induced a change in the mycorrhizal partners of *Kobresia pygmaea*. Arbuscular mycorrhizal fungi, capable of capturing nutrients released by other microorganisms’ hydrolytic enzymes, increased along the sequence of increasing hydrolytic organic matter decomposition (S0–S2) and optimized the access to otherwise rapidly leached or consumed ammonia and nitrate. However, toward stage S4, there was a shift in favor of ectomycorrhizal partners of *Kobresia pygmaea*, being specialized for the active mobilization of organic matter-bound nutrients using their own oxidative enzymes. The concurrent decrease in urease activity from S3 to S4, reflecting successive depletion of the easily accessible N in the soil, is evidence of a shift in ecosystem nutrition strategy: mineral nutrient release through AMF-mediated and prokaryote-driven hydrolysis was replaced by fungi-based nutrient scavenging for nutritional elements “locked up” in complex structural root-mat tissues using oxidative enzymes. Such fundamental shifts in nutritional strategies and biogeochemical cycles suggest that degradation will have large-scale feedback on multiple environmental processes.

As a result of organic matter decomposition, we expected kinetic isotope fractionation to induce a continuous δ^13^C increase along the degradation gradient^23^. However, the opposite was found for early degradation stages. δ^13^C values decreased from S0 to S4 within the upper 20 cm, while δ^13^C values increased from S4 to S5. We attribute the δ^13^C decrease (S0–S4) to a relative accumulation of more complex, ^13^C-depleted organic compounds such as lignin^24^. Assuming a 4.3‰ depletion in lignin δ^13^C compared to bulk plant material^24^ and a recovery of 38% of the lignin C by the vanillyl, syringyl, cinnamyl method^25^, the relative increase of lignin from 0.9 to 4.7% of SOC accounts for nearly 23% of the observed δ^13^C shift across the degradation sequence. However, lignin is only one compound class among others (cutins, suberins, etc.) with complex structures and depleted δ^13^C values. Lipids have even more depleted δ^13^C values than lignin^24^. The negative δ^13^C shifts observed with soil degradation from S0 to S4 likely reflect the relative accumulation of these rather persistent compounds in addition to lignin. The intact *Kobresia* root mats at the research site consist of about 6 kg root dry matter m^−2^^5^, including many woody roots that contain large amounts of lignin and suberin. Root dieback during soil degradation, therefore, provides a substantial source of complex, ^13^C-depleted compounds. Relative lignin enrichment of the SOC pool was evident not only in the δ^13^C decrease but also in the increased abundance of fungal and bacterial specialists decomposing complex SOM (e.g., *Rhizobiales* and *Agaricomycetes*). The elevated abundance of these groups was accompanied by increased phenoloxidase activity, which catalyzes the nonspecific oxidation of complex organic substances, especially lignin. Consequently, after easily available C pools are depleted (S3), complex organic compounds are successively degraded as microbial communities adapt to these distinct carbon sources. While microbial communities changed gradually from S0 to S3, a profound shift in community structure and functions occurred from S3 to S4 (evidenced by a vertical shift in direction in the NMDS plots; Supplementary Figs. 6 and 8). This transition coincided with (a) the exhaustion of easily accessible, hydrolyzable N sources, (b) the complete loss of the topsoil through mineralization and erosion, and (c) the first establishment of pioneer plants on the bare subsoil between *Kobresia* root-mat remnants. The new fungal symbionts of these pioneer plants explain the particularly pronounced shift in the fungal community from S3 to S4, representing this new ecosystem state. Degradation exceeding S4, therefore, constitutes a severe alteration of microbial community composition and functioning, beyond which biotic and abiotic ecosystem properties have changed so severely that ecosystem recovery is unlikely.

Taken together, overgrazing of *Kobresia* pastures on the TP induces a highly interrelated sequence of abiotic and biotic degradation processes. Polygonal crack extension and root-mat dieback as a result of freeze-thaw cycles and overgrazing induce topsoil erosion and progressive SOC decomposition and N loss. This is accompanied by a shift in microbial community structure and functions to adapt to the altered availability of SOC and N. Studies of the past decade predict an intensification of the degradation of this pastoral ecosystem because of the large population that is dependent on livestock products^3^, increasing stocking rates close to settlements^4^ and diminishing availability of high-quality grazing grounds following degradation^9^. Once the critical point between S3 and S4 has been passed, conventional strategies for mitigating degradation are unlikely to prevent complete loss of the entire C-rich topsoil, with cascading effects on microbial functioning and the related ecosystem services.

The challenges brought about by TP degradation can only be addressed through lowering livestock densities, and perhaps more importantly increasing livestock mobility to maintain important functions of the vulnerable *Kobresia* ecosystem. In 2011, China proposed the Ecological Conservation Redline strategy that protects ecologically fragile zones like the TP^26^ by prioritizing and prohibiting rangeland degradation. Tibetan pastoralists increasingly manage their rangelands through contracting, assessing limits to livestock holdings based on land availability^27^, e.g., by “multi-household management pattern”. This practice has been shown to reduce SOC losses considerably^28^, more closely resembling pre-settlement grassland management. Further, the reintroduction of seasonal livestock grazing in the framework of community-based villages can protect or even restore vegetation and soil^29^, provided that a certain degradation stage has not been surpassed. Once this critical point is crossed, reseeding has been proposed as a restoration strategy^30^. However, reseeding of heavily degraded areas generally requires N and P fertilization^30^, which carries the risk of nutrient leaching and headwater pollution and is nevertheless often unsuccessful^31^. Dong et al.^31^ studied the restoration potential of TP grasslands and their soil chemical and physical properties in three depth increments (0–4, 4–10, and 10–20 cm). The results show that neither the decreased water holding capacity, nor the SOC and N content could be restored in the reestablished artificial grasslands this was attributed to the loss of the topsoil, changed soil texture, and changes in vegetation composition.

This demonstrates the threshold behavior of TP’s pastures, where a complete recovery from highly degraded stages is unlikely, due to slow pedogenic processes and vegetation restoration, as well as continuously increasing global warming.

## Methods

### Literature study

Literature considering the effect of pasture degradation on SOC, N, and clay content, as well as bulk density (BD), was assembled by searching (i) Web of Science V.5.22.1, (ii) ScienceDirect (Elsevier B.V.) (iii) Google Scholar, and (iv) the China Knowledge Resource Integrated Database (CNKI). Search terms were “degradation gradient”, “degradation stages”, “alpine meadow”, “Tibetan Plateau”, “soil”, “soil organic carbon”, and “soil organic matter” in different combinations. The criteria for including a study in the analysis were: (i) a clear and comprehensible classification of degradation stages was presented, (ii) data on SOC, N, and/or BD were reported, (iii) a non-degraded pasture site was included as a reference to enable an effect size analysis and the calculation of SOC and N losses, (iv) sampling depths and study location were clearly presented. (v) Studies were only considered that took samples in 10 cm depth intervals, to maintain comparability to the analyses from our own study site. The degradation stages in the literature studies were regrouped into the six successive stages (S0–S5) according to the respective degradation descriptions. In total, we compiled the results of 49 publications published between 2002 and 2020.

When SOM content was presented, this was converted to SOC content using a conversion factor of 2.0^32^. SOC and N stocks were calculated using the following equation:

1 $${{{{{\rm{Elemental\; stock}}}}}}=100* {{{{{\rm{content}}}}}}* {{{{{\rm{BD}}}}}}* {{{{{\rm{depth}}}}}}$$

where elemental stock is SOC or N stock [kg ha^−1^]; content is SOC or N content [g kg^−1^]; BD is soil bulk density [g cm^−3^] and depth is the soil sampling depth [cm].

The effect sizes of individual variables (i.e., SOC and N stocks as well as BD) were quantified as follows:

2 $${{{{{\rm{ES}}}}}}=\,\frac{(D-R)}{R* 100 \% }$$

where *ES* is the effect size in %, *D* is the value of the corresponding variable in the relevant degradation stage and *R* is the value of each variable in the non-degraded stage (reference site). When *ES* is positive, zero, or negative, this indicates an increase, no change, or decrease, respectively, of the parameter compared to the non-degraded stage.

### Experimental design of the field study

Large areas in the study region are impacted by grassland degradation. In total, 45% of the surface area of the Kobresia pasture ecosystem on the TP is already degraded^2^. The experiment was designed to differentiate and quantify SOC losses by erosion vs. net decomposition and identify underlying shifts in microbial community composition and link these to changes in key microbial functions in the soil C cycle. We categorized the range of *Kobresia* root-mat degradation from non-degraded to bare soils into six successive degradation stages (S0–S5). Stage S0 represented non-degraded root mats, while stages S1–S4 represented increasing degrees of surface cracks, and bare soil patches without root mats defined stage S5 (Supplementary Fig. 1). All six degradation stages were selected within an area of about 4 ha to ensure equal environmental conditions and each stage was sampled in four field replicates. However, the studied degradation patterns are common for the entire *Kobresia* ecosystem (Supplementary Fig. 1).

### Site description

The field study was conducted near Nagqu (Tibet, China) in the late summer 2013 and 2015. The study site of about 4 ha (NW: 31.274748°N, 92.108963°E; NE: 31.274995°N, 92.111482°E; SW: 31.273488°N, 92.108906°E; SE: 31.273421°N, 92.112025°E) was located on gentle slopes (2–5%) at 4,484 m a.s.l. in the core area of the *Kobresia pygmaea* ecosystem according to Miehe et al.^8^. The vegetation consists mainly of *K. pygmaea*, which covers up to 61% of the surface. Other grasses, sedges, or dwarf rosette plants (*Carex ivanoviae, Carex* spp*., Festuca* spp*., Kobresia pusilla, Poa* spp*., Stipa purpurea, Trisetum* spp.) rarely cover more than 40%. The growing season is strongly restricted by temperature and water availability. At most, it lasts from mid-May to mid-September, but varies strongly depending on the onset and duration of the summer monsoon. Mean annual precipitation is 431 mm, with roughly 80% falling as summer rains. The mean annual temperature is −1.2 °C, while the mean maximum temperature of the warmest month (July) is +9.0 °C^2^.

A characteristic feature of *Kobresia* pastures is their very compact root mats, with an average thickness of 15 cm at the study site. These consist mainly of living and dead *K. pygmaea* roots and rhizomes, leaf bases, large amounts of plant residue, and mineral particles. Intact soil is a Stagnic Eutric Cambisol (Humic), developed on a loess layer overlying glacial sediments and containing 50% sand, 33% silt, and 17% clay in the topsoil (0–25 cm). The topsoil is free of carbonates and is of neutral pH (pH in H_2_O: 6.8)^5^. Total soil depth was on average 35 cm.

The site is used as a winter pasture for yaks, sheep, and goats from January to April. Besides livestock, large numbers of plateau pikas (*Ochotona*) are found on the sites. These animals have a considerable impact on the plant cover through their burrowing activity, in particular the soil thrown out of their burrows, which can cover and destroy the *Kobresia* turf.

### Sampling design

The vertical and horizontal extent of the surface cracks was measured for each plot (Supplementary Table 2). Vegetation cover was measured and the aboveground biomass was collected in the cracks (Supplementary Table 2). In general, intact *Kobresia* turf (S0) provided high resistance to penetration as measured by a penetrologger (Eijkelkamp Soil and Water, Giesbeek, NL) in 1 cm increments and four replicates per plot.

Soil sampling was conducted using soil pits (30 cm length × 30 cm width × 40 cm depth). Horizons were classified and then soil and roots were sampled for each horizon directly below the cracks. Bulk density and root biomass were determined in undisturbed soil samples, using soil cores (10 cm height and 10 cm diameter). Living roots were separated from dead roots and root debris by their bright color and soft texture using tweezers under magnification, and the roots were subsequently washed with distilled water to remove the remaining soil. Because over 95% of the roots occurred in the upper 25 cm^5^, we did not sample for root biomass below this depth.

Additional soil samples were taken from each horizon for further analysis. Microbial community and functional characterization were performed on samples from the same pits but with a fixed depth classification (0–5 cm, 5–15 cm, 15–35 cm) to reduce the number of samples.

### Plant and soil analyses

Soil and roots were separated by sieving (2 mm) and the roots subsequently washed with distilled water. Bulk density and root density were determined by dividing the dry soil mass (dried at 105 °C for 24 h) and the dry root biomass (60 °C) by the volume of the sampling core. To reflect the root biomass, root density was expressed per soil volume (mg cm^−3^). Soil and root samples were milled for subsequent analysis.

### Elemental concentrations and SOC characteristics

Total SOC and total N contents and stable isotope signatures (δ^13^C and δ^15^N) were analyzed using an isotope ratio mass spectrometer (Delta plus, Conflo III, Thermo Electron Cooperation, Bremen, Germany) coupled to an elemental analyzer (NA 1500, Fisons Instruments, Milano, Italy). Measurements were conducted at the Centre for Stable Isotope Research and Analysis (KOSI) of the University of Göttingen. The δ^13^C and δ^15^N values were calculated by relating the isotope ratio of each sample (R_sample_ = ^13^C/^12^C or ^15^N/^14^N) to the international standards (Pee Dee Belemnite ^13^C/^12^C ratio for δ^13^C; the atmospheric ^15^N/^14^N composition for δ^15^N).

Soil pH of air-dried soil was measured potentiometrically at a ratio (v/v) of 1.0:2.5 in distilled water.

Lignin phenols were depolymerized using the CuO oxidation method^25^ and analyzed with a gas chromatography-mass spectrometry (GC–MS) system (GC 7820 A, MS 5977B, Agilent Technologies, Waldbronn, Germany). Vanillyl and syringyl units were calculated from the corresponding aldehydes, ketones, and carboxylic acids. Cinnamyl units were derived from the sum of *p*-coumaric acid and ferulic acid. The sum of the three structural units (VSC = V + S + C) was considered to reflect the lignin phenol content in a sample.

### DNA extraction and PCR

Samples were directly frozen on site at −20 °C and transported to Germany for analysis of microbial community structure. Total DNA was extracted from the soil samples with the PowerSoil DNA isolation kit (MoBio Laboratories Inc., Carlsbad, CA, USA) according to the manufacturer’s instructions, and DNA concentration was determined using a NanoDrop 1000 spectrophotometer (Thermo Fisher Scientific, Wilmington, DE, USA). The extracted DNA was amplified with forward and reverse primer sets suitable for either t-RFLP (fluorescence marked, FAM) or Illumina MiSeq sequencing (Illumina Inc., San Diego, USA): V3 (5’-CCT ACG GGN GGC WGC AG-3’) and V4 (5’-GAC TAC HVG GGT ATC TAA TCC-3’) primers were used for bacterial 16 S rRNA genes whereas ITS1 (5’-CTT GGT CAT TTA GAG GAA GTA A-3’), ITS1-F_KYO1 (5’-CTH GGT CAT TTA GAG GAA STA A-3’), ITS2 (5’-GCT GCG TTC TTC ATC GAT GC-3’) and ITS4 (5’-TCC TCC GCT TAT TGA TAT GC-3’) were used for fungi^33,34^. Primers for Illumina MiSeq sequencing included adaptor sequences (forward: 5’-TCG TCG GCA GCG TCA GAT GTG TAT AAG AGA CAG-3’; reverse: 5’-GTC TCG TGG GCT CGG AGA TGT GTA TAA GAG ACA G-3’)^33^. PCR was performed with the Phusion High-Fidelity PCR kit (New England Biolabs Inc., Ipswich, MA, USA) creating a 50 µl master mix with 28.8 µl H_2_O_molec_, 2.5 µl DMSO, 10 µl Phusion GC buffer, 1 µl of forward and reverse primer, 0.2 µl MgCl_2_, 1 µl dNTPs, 0.5 µl Phusion HF DNA Polymerase, and 5 µl template DNA. PCR temperatures started with initial denaturation at 98 °C for 1 min, followed by denaturation (98 °C, 45 s), annealing (48/60 °C, 45 s), and extension (72 °C, 30 s). These steps were repeated 25 times, finalized again with a final extension (72 °C, 5 min), and cooling to 10 °C. Agarose gel electrophoresis was used to assess the success of the PCR and the amount of amplified DNA (0.8% gel:1.0 g Rotigarose, 5 µl Roti-Safe Gelstain, Carl Roth GmbH & Co. KG, Karlsruhe, Germany; and 100 ml 1× TAE-buffer). PCR product was purified after initial PCR and restriction digestion (t-RFLP) with either NucleoMag 96 PCR (16 S rRNA gene amplicons, Macherey-Nagel GmbH & Co. KG, Düren, Germany) or a modified clean-up protocol after Moreau (t-RFLP)^35^: 3× the volume of the reaction solution as 100% ethanol and ¼x vol. 125 mM EDTA was added and mixed by inversion or vortex. After incubation at room temperature for 15 min, the product was centrifuged at 25,000 × *g* for 30 min at 4 °C. Afterwards the supernatant was removed, and the inverted 96-well plate was centrifuged shortly for 2 min. Seventy microliters ethanol (70%) were added and centrifuged at 25,000 × *g* for 30 min at 4 °C. Again, the supernatant was removed, and the pallet was dried at room temperature for 30 min. Finally, the ethanol-free pallet was resuspended in H_2_O_molec_.

### T-RFLP fingerprinting

The purified fluorescence-labeled PCR products were digested with three different restriction enzymes (*MspI* and *BstUI, HaeIII*) according to the manufacturer’s guidelines (New England Biolabs Inc., Ipswich, MA, USA) with a 20 µl master mix: 16.75 µl H_2_O_molec_, 2 µl CutSmart buffer, 0.25 or 0.5 µl restriction enzyme, and 1 µl PCR product for 15 min at 37 °C (*MspI*) and 60 °C (*BstUI, HaeIII*), respectively. The digested PCR product was purified a second time^35^, dissolved in Super-DI Formamide (MCLAB, San Francisco, CA, USA) and, along with Red DNA size standard (MCLAB, San Francisco, USA), analyzed in an ABI Prism 3130 Genetic Analyzer (Applied Biosystems, Carlsbad, CA, USA). Terminal restriction fragments shorter than 50 bp and longer than 800 bp were removed from the t-RFLP fingerprints.

### 16 S rRNA gene and internal transcribed spacer (ITS) sequencing and sequence processing

The 16 S rRNA gene and ITS paired-end raw reads for the bacterial and fungal community analyses were deposited in the National Center for Biotechnology Information (NCBI) Sequence Read Archive (SRA) and can be found under the BioProject accession number PRJNA626504. This BioProject contains 70 samples and 139 SRA experiments (SRR11570615–SRR11570753) which were processed using CASAVA software (Illumina, San Diego, CA, USA) for demultiplexing of MiSeq raw sequences (2 × 300 bp, MiSeq Reagent Kit v3).

Paired-end sequences were quality-filtered with fastp (version 0.19.4)^36^ using default settings with the addition of an increased per base phred score of 20, base-pair corrections by overlap (-c), as well as 5′- and 3′-end read trimming with a sliding window of 4, a mean quality of 20 and minimum sequence size of 50 bp. Paired-end sequences were merged using PEAR v0.9.11^37^ with default parameters. Subsequently, unclipped reverse and forward primer sequences were removed with cutadapt v1.18^38^ with default settings. Sequences were then processed using VSEARCH (v2.9.1)^39^. This included sorting and size-filtering (—sortbylength,—minseqlength) of the paired reads to ≥300 bp for bacteria and ≥140 bp for ITS1, dereplication (—derep_fulllength). Dereplicated sequences were denoised with UNOISE3^40^ using default settings (—cluster_unoise—minsize 8) and chimeras were removed (—uchime3_denovo). An additional reference-based chimera removal was performed (—uchime_ref) against the SILVA^41^ SSU NR database (v132) and UNITE^42^ database (v7.2) resulting in the final set of amplicon sequence variants (ASVs)^43^. Quality-filtered and merged reads were mapped to ASVs (—usearch_global–id 0.97). Classification of ASVs was performed with BLAST 2.7.1+ against the SILVA SSU NR (v132) and UNITE (v7.2) database with an identity of at least 90%. The ITS sequences contained unidentified fungal ASVs after UNITE classification, these sequences were checked (blastn)^44^ against the “nt” database (Nov 2018) to remove non-fungal ASVs and only as fungi classified reads were kept. Sample comparisons were performed at the same surveying effort, utilizing the lowest number of sequences by random selection (total 15,800 bacteria, 20,500 fungi). Species richness, alpha and beta diversity estimates, and rarefaction curves were determined using the QIIME 1.9.1^45^ script *alpha_rarefaction.py*.

The final ASV tables were used to compute heatmaps showing the effect of degradation on the community using R (Version 3.6.1, R Foundation for Statistical Computing, Vienna, Austria) and R packages “gplots”, “vegan”, “permute” and “RColorBrewer”. Fungal community functions were obtained from the FunGuild database^46^. Plant mycorrhizal association types were compiled from the literature^38–41,47–50^. If no direct species match was available, the mycorrhizal association was assumed to remain constant within the same genus.

### Enzyme activity

Enzyme activity was measured to characterize the functional activity of the soil microorganisms. The following extracellular enzymes, involved in C, N, and P transformations, were considered: two hydrolases (β-glucosidase and xylanase), phenoloxidase, urease, and alkaline phosphatase. Enzyme activities were measured directly at the sampling site according to protocols after Schinner et al.^51^. Beta-glucosidase was incubated with saligenin for 3 h at 37 °C, xylanase with glucose for 24 h at 50 °C, phenoloxidase with L-3,4-dihydroxy phenylalanine (DOPA) for 1 h at 25 °C, urease with urea for 2 h at 37 °C and alkaline phosphatase on P-nitrophenyl phosphate for 1 h at 37 °C. Reaction products were measured photometrically at recommended wavelengths (578, 690, 475, 660, and 400 nm, respectively).

### SOC stocks and SOC loss

The SOC stocks (in kg C m^−2^) for the upper 30 cm were determined by multiplying the SOC content (g C kg^−1^) by the BD (g cm^−3^) and the thickness of the soil horizons (m). SOC losses (%) were calculated for each degradation stage and horizon and were related to the mean C stock of the reference stage (S0). The erosion-induced SOC loss of the upper horizon was estimated by considering the topsoil removal (extent of vertical soil cracks) of all degraded soil profiles (S1–S5) and the SOC content and BD of the reference (S0). To calculate the mineralization-derived SOC loss, we accounted for the effects of SOC and root mineralization on both SOC content and BD. Thus, we used the SOC content and BD from each degradation stage (S1–S5) and multiplied it by the mean thickness of each horizon (down to 30 cm) from the reference site (S0). The disentanglement of erosion-derived SOC loss from mineralization-derived SOC loss was based on explicit assumptions that (i) erosion-derived SOC losses are mainly associated with losses from the topsoil, and (ii) the decreasing SOC contents in the erosion-unaffected horizons were mainly driven by mineralization and decreasing root C input.

### Statistical analyses

Statistical analyses were performed using PASW Statistics (IBM SPSS Statistics) and R software (Version 3.6.1). Soil and plant characteristics are presented as means and standard errors (means ± SE). The significance of treatment effects (S0–S5) and depth was tested by one-way ANOVA at *p* < 0.05. Prior to this, we checked for normality and homogeneity of variance using the Shapiro–Wilk test and Levene’s test, respectively.

Post-hoc multiple comparisons were carried out using the LSD or Tukey HSD ANOVA, if normality was indicated. In cases of non-normal distribution, the nonparametric Kruskal–Wallis test was implemented coupled with a Bonferroni correction. To detect relationships between various plant and soil characteristics, we used linear and nonlinear regressions. Correlations were deemed significant for single regressions at *p* < 0.05.

Before testing for significant differences, three outliers were detected by Grubbs outlier test (*p* < 0.05) in the bacterial t-RFLP and MiSeq datasets: MspI: S0_B_0-5, S1_E_0-5; BstuI: S5_A_15-35; MiSeq: S3_E_5-15 and were excluded from the analysis. T-RFLP and MiSeq data of all degradation stages were compared for significant differences with MANOVA, based on the Bray–Curtis index for dissimilarity. For pairwise multilevel comparisons, “pairwiseAdonis” was used^52^. Differences in microbial community data from t-RFLP and MiSeq were displayed in non-metric multidimensional scaling (NMDS) plots and environmental factors were correlated by canonical correspondence analysis (CCA). Statistics on community data were carried out with R statistical software (Version 3.6.1).

### Reporting summary

Further information on research design is available in the Nature Research Reporting Summary linked to this article.

## Supplementary information

Supplementary Information

Reporting Summary

## Data Availability

The data generated in this study have been deposited in the PANGAEA Open Access library under accession code 10.1594/PANGAEA.918249. The 16 S rRNA and ITS gene paired-end raw reads for the bacterial and fungal community analyses have been deposited in the National Center for Biotechnology In-formation (NCBI) Sequence Read Archive (SRA) under the accession code PRJNA626504; BioProject: Tibetan plateau microbiome.
