# Supplementary material for: Microbial functional changes mark irreversible course of Tibetan grassland degradation
Source: Nat Commun. 2022 May 13;13:2681. doi: 10.1038/s41467-022-30047-7 (PMC9106683; doi:10.1038/s41467-022-30047-7)
Supplement: Supplementary file 1 — Supplementary Information [file 41467_2022_30047_MOESM1_ESM.pdf]

Supplementary information for microbial functional changes mark irreversible course of Tibetan grassland degradation

Authors

Andreas Breidenbach[1,2†,] Per-Marten Schleuss[3†], Shibin Liu[4], Dominik Schneider[5], Michaela A. Dippold[1,2], Tilman de la Haye[6], Georg Miehe[7], Felix Heitkamp[8], Elke Seeber[9], Kyle Mason-Jones[10], Xingliang Xu[11,12], Yang Huanming[13], Jianchu Xu[14], Tsechoe Dorji[12,15], Matthias Gube[16], Helge Norf[17], Jutta Meier[18], Georg Guggenberger[19,20], Yakov Kuzyakov[21,22], Sandra Spielvogel[6*]

Supplementary Materials

Supplementary Figure 1

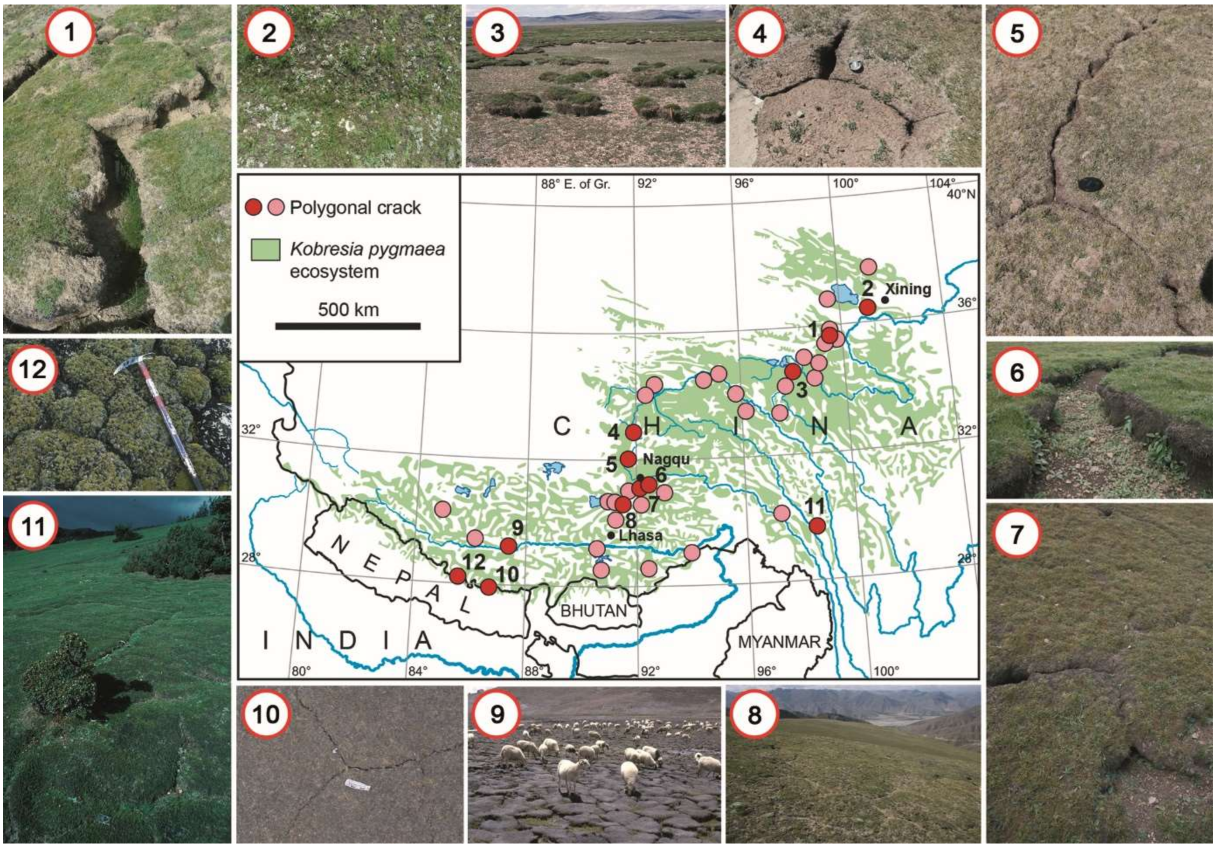

Supplementary Figure 1. **Selected examples of polygonal cracking and extension on the Tibetan Plateau (TP).** Red dots show locations of polygonal cracks based on observations during field trips on the TP and the Himalayas between 1982 and 2015. The map reflects the widespread distribution of this specific degradation phenomenon, but not its actual distribution or intensity. Photo credits: E. Seeber (1, 2), G. Miehe (3–5, 9, 11, 12), P.-M. Schleuss (6–8) and R. Bäumler (10). Cartography: C. Enderle.

Supplementary Figure 2

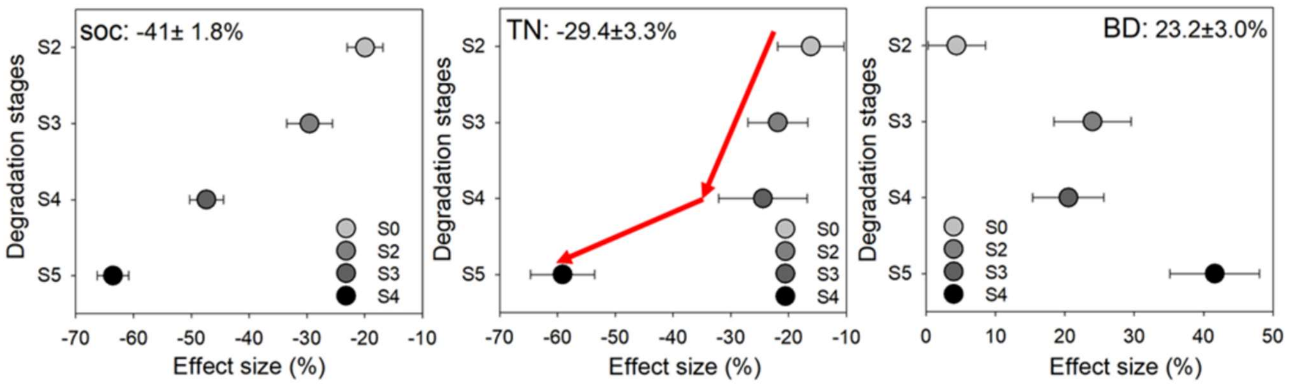

Supplementary Figure 2. **Effect sizes of SOC (soil organic carbon) content, total nitrogen (TN) content and soil bulk density (BD) for degradation stages S1 to S4 compared to non-degraded pastures (S0).** The percentage value at the top shows the average effect size of the four degradation stages (meta-analysis including 594 single observations from literature studies published between 2002 and 2020, error bars display standard error).

Supplementary Figure 3.

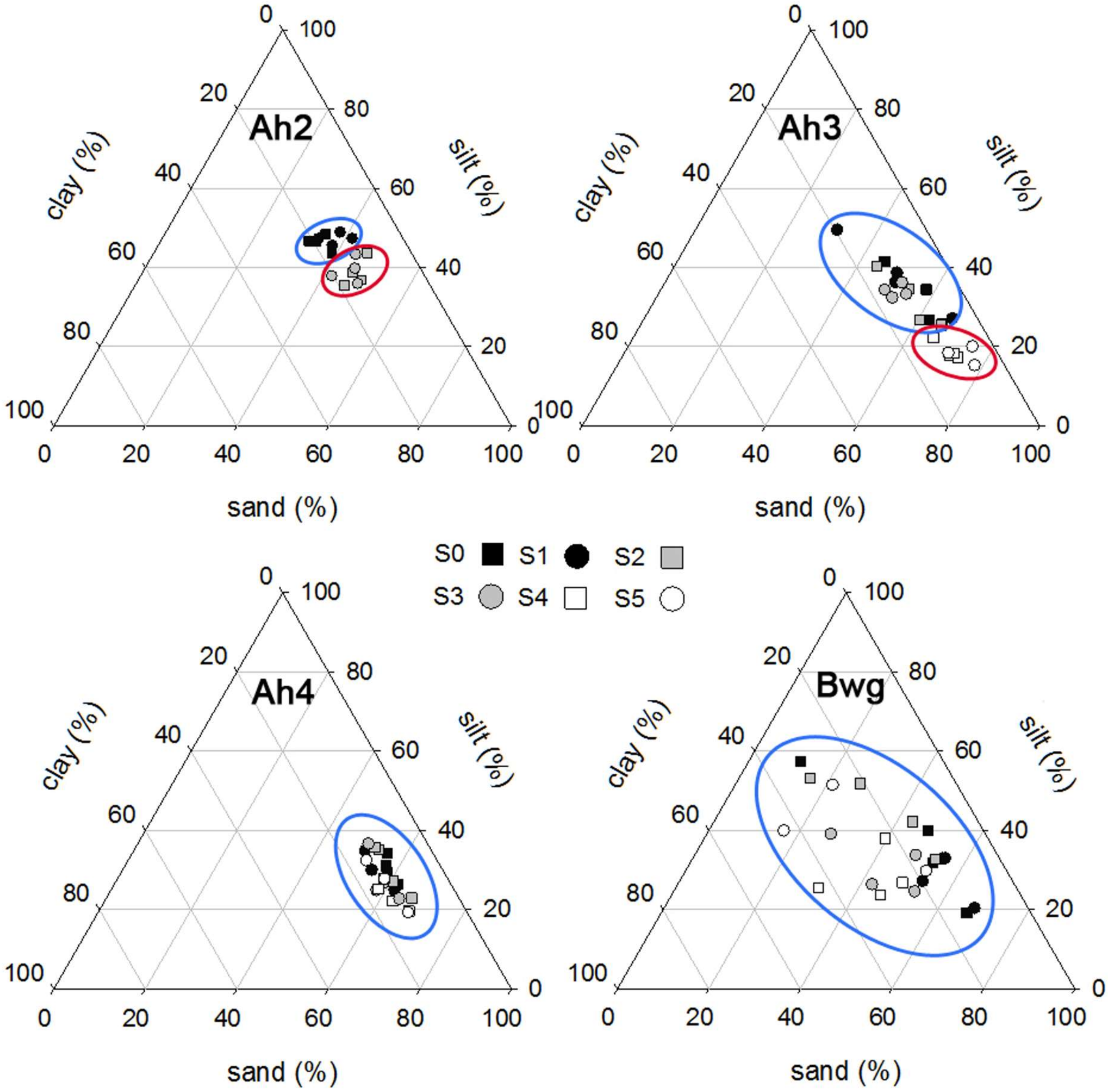

Supplementary Figure 3. **Changes in particle size distribution for each soil horizon at each degradation stage**. The soil texture was measured for each soil horizon (Ah2, Ah3, Ah4, Bwg), except for the densely rooted and thin Ah1 horizon, with low amounts of mineral soil. Circled in blue are the “protected horizons” having an overlying horizon. Circled in red are the upper soil horizons, which are prone to erosion processes and shift toward a coarser soil texture.

Supplementary Figure 4

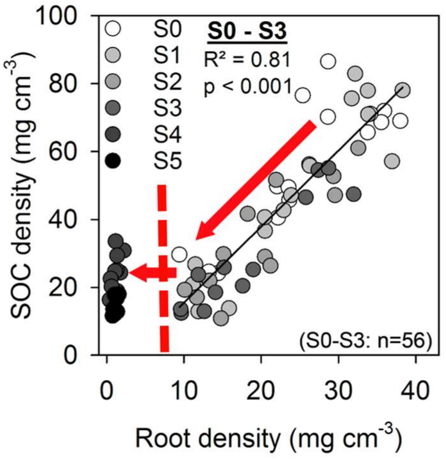

Supplementary Figure 4. **Relationship between soil organic carbon (SOC) density and root density for all soil horizons at each degradation stage.** Linear regression covers degradation stages S0 to S3. At degradation stages S4 and S5, SOC density is independent of root density and represents remnant SOC.

Supplementary Figure 5

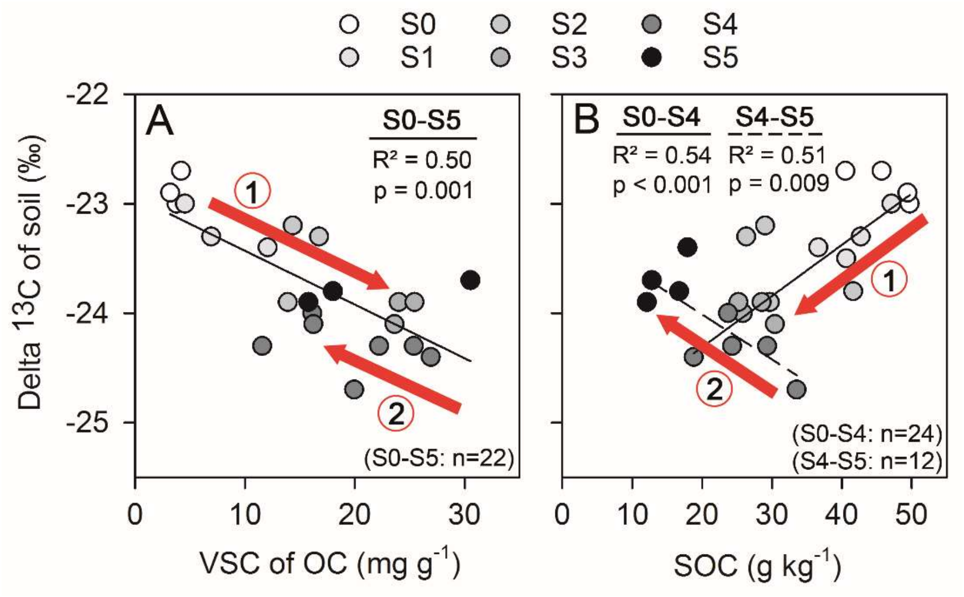

Supplementary Figure 5. **Relationship between δ^{13}C values and (A) content of lignin monomers vanillyl, syringyl, and cinnamyl (VSC) or (B) soil organic carbon (SOC) content in the Ah3 horizon for each degradation stage.** The pattern along the degradation sequence can be explained by the following processes: (1) decreasing SOC concentrations associated with a relative accumulation of lignin and an associated decrease in δ$^{13}C_{SOC}$ values (S0–S3) and (2) decreasing SOC concentrations associated with lignin degradation and $^{13}C_{SOC}$ enrichment (S4, S5).

Supplementary Figure 6

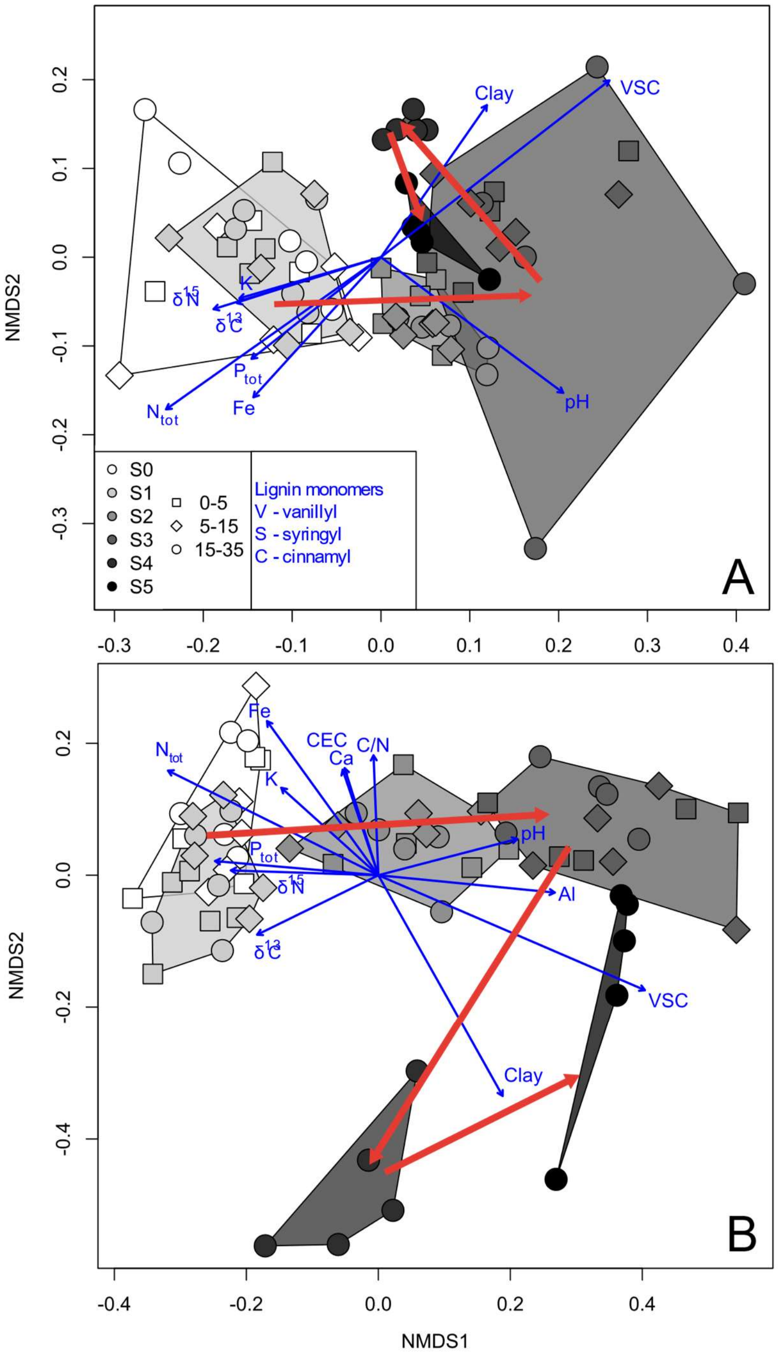

Supplementary Figure 6. **NMDS (non-metric multidimensional scaling) plots derived from t-RFLP data for the bacterial (A) and fungal (B) communities.** Shaded areas mark each degradation stage, symbols indicate soil depth, and blue arrows show significantly correlated abiotic factors (canonical correspondence analysis, $p < 0.05$). Red arrows mark key structural shifts of the microbial community.

Supplementary Figure 7

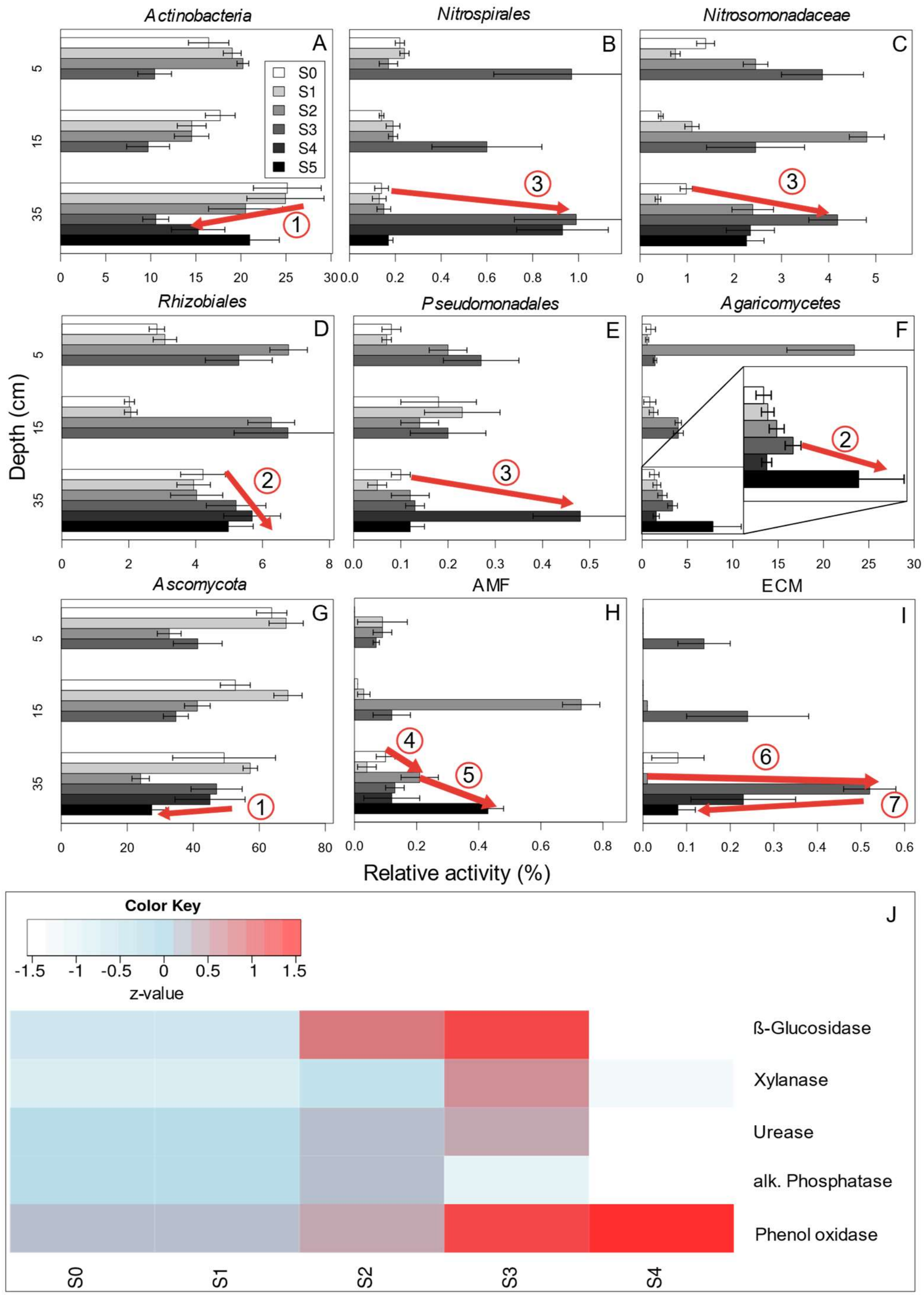

Supplementary Figure 7. **Relative abundance of key microbial functional groups.** Shifts are indicated by red arrows for the lowest depth, representing the complete range of degradation stages (S0–S5): (1) decline of bacterial (**A**) and fungal (**G**) litter degraders, (2) increase in bacterial (**D**) and fungal (**F**) litter degraders, (3) increase in nitrifying (**B, C**) and denitrifying (**E**) bacteria, (4) increase in arbuscular mycorrhizal fungi (AMF) until S2 (**H**), (6) which are then replaced by ectomycorrhizal fungi (ECM) (**I**); (7) ECM decline toward S5 (**I**) as new plants with new AMF (5) become established after *Kobresia pygmaea* disappears (**H**). Key enzyme activities (**J**) reflect shifts in microbial community functions from hydrolytic to oxidative soil organic carbon (SOC) decomposition.

Supplementary Figure 8

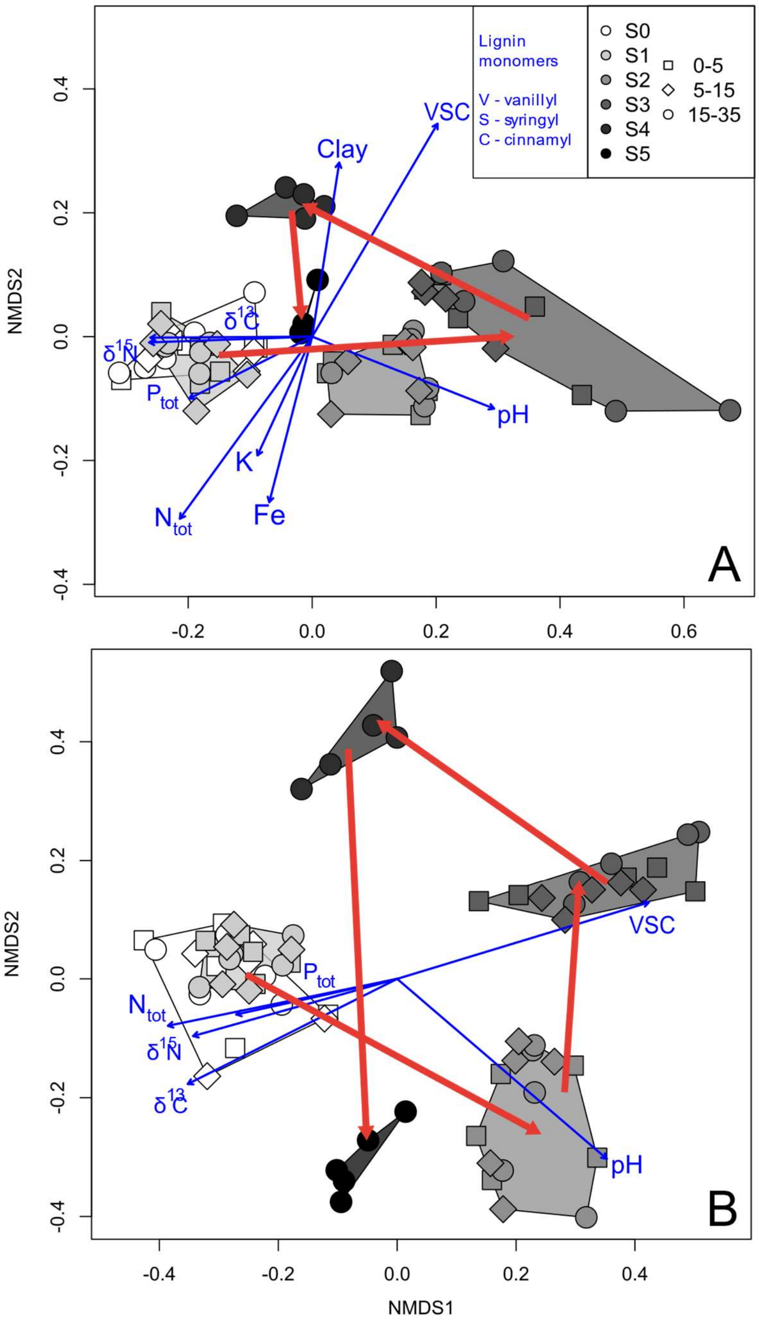

Supplementary Figure 8. **NMDS (non-metric multidimensional scaling) plots derived from Illumina MiSeq sequencing data of the bacterial (A) and fungal (B) communities along degradation stages.** Shaded areas mark each degradation stage, symbols indicate soil depth, and blue arrows show significantly correlated abiotic factors (by CCA, canonical-correlation analysis).

Supplementary Figure 9

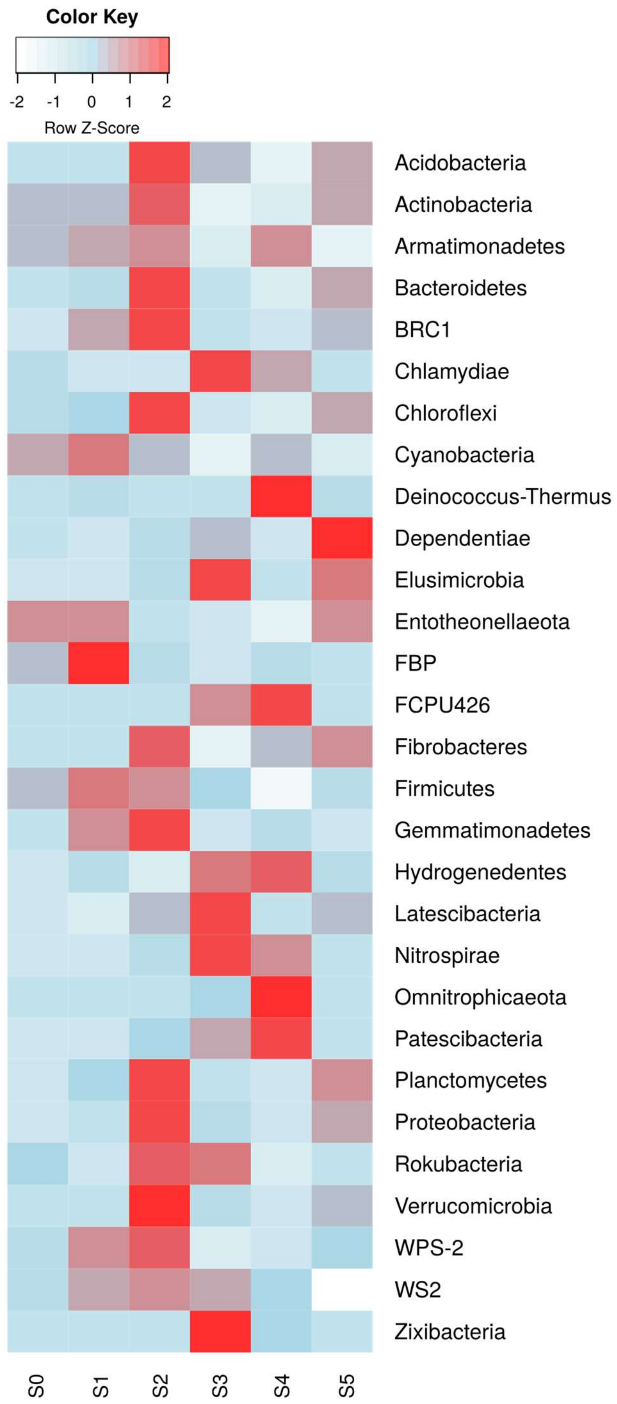

Supplementary Figure 9. **Heat map of the bacterial community along degradation stages (soil depth 15–35 cm).** Shades of color indicate abundance of bacterial phyla as z-values.

Supplementary Figure 10a

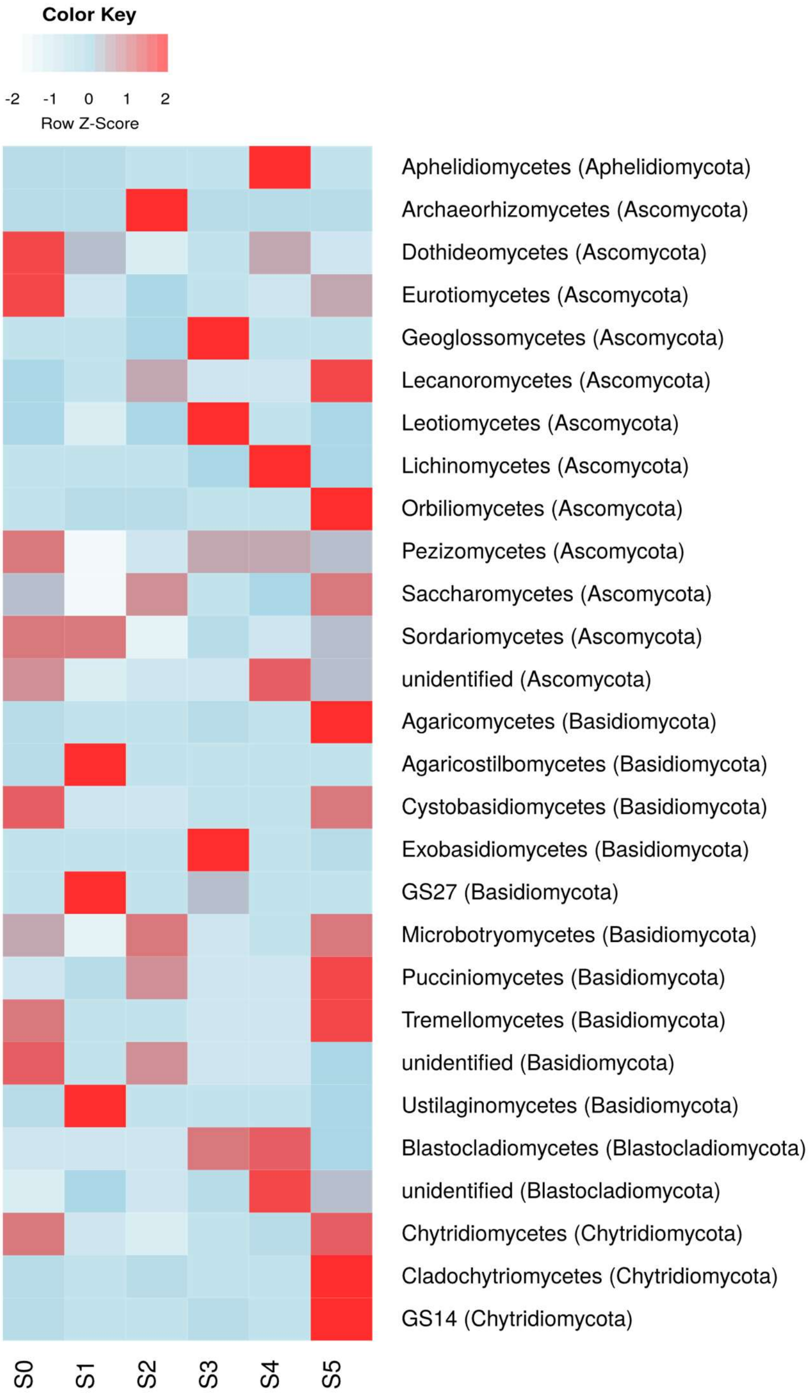

Supplementary Figure 10a. **Heat map of the fungal community along degradation stages (soil depth 15–35 cm).** Shades of color indicate abundance of fungal phyla as z-values.

Supplementary Figure 10b

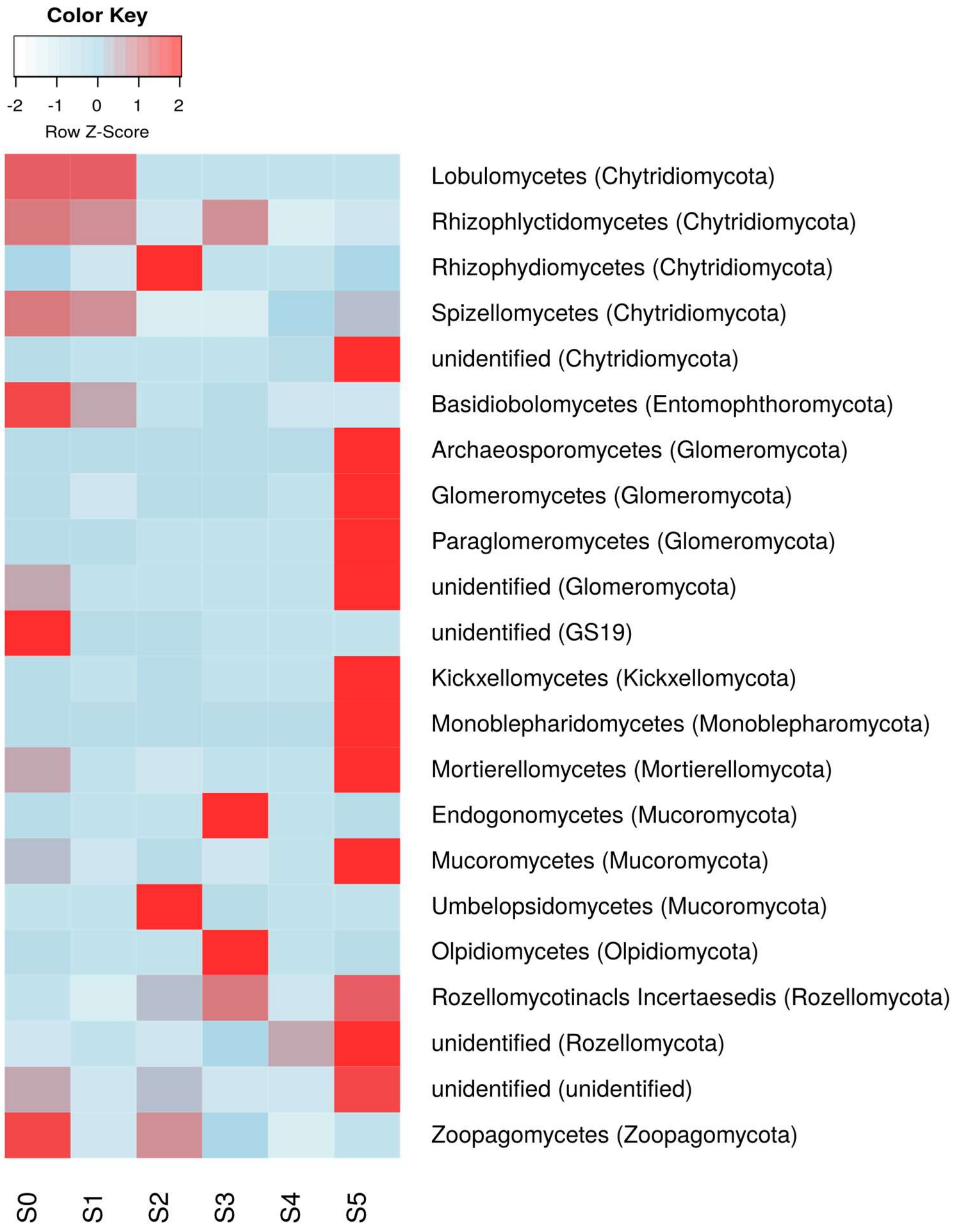

Supplementary Figure 10b. **Heat map of the fungal community along degradation stages (soil depth 15–35 cm).** Shades of color indicate abundance of fungal phyla as z-values (continuation from S10a).

Supplementary Figure 11

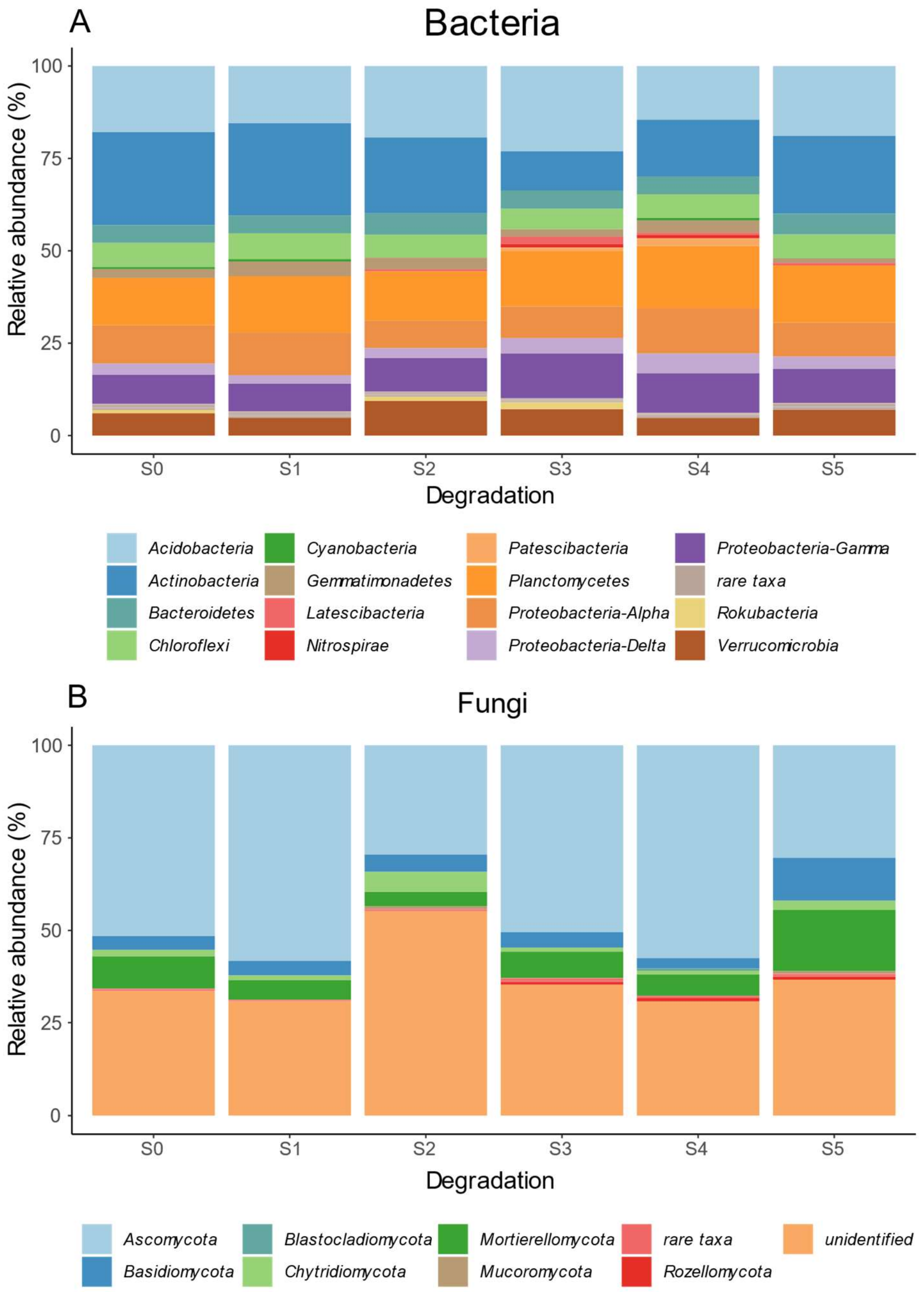

Supplementary Figure 11. **Relative abundance of bacterial (A) and fungal (B) phyla found along the degradation sequence (soil depth 15–35 cm).** Phyla amounting to less than 0.5% were grouped as ‘rare taxa’.

Supplementary Figure 12

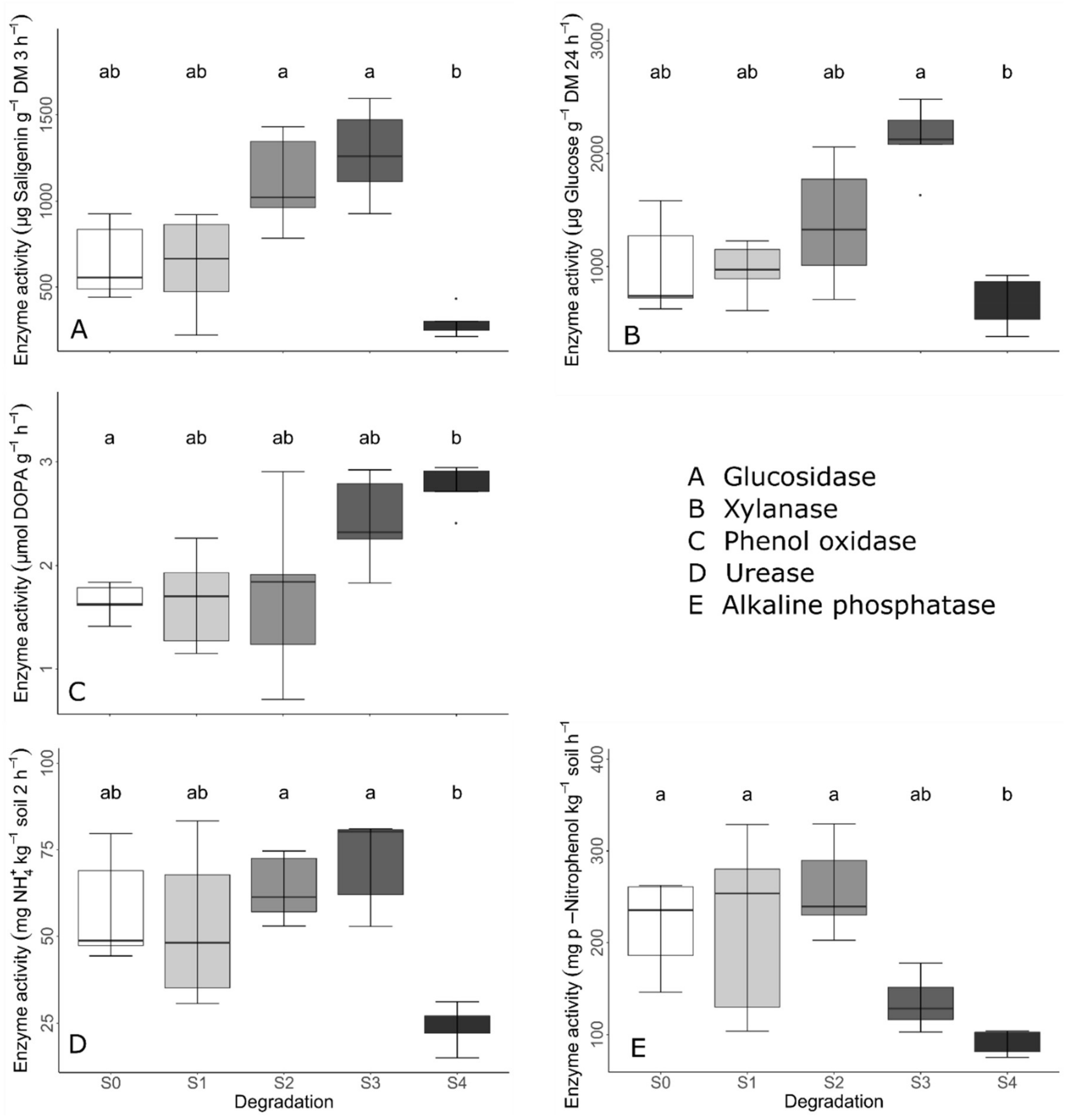

Supplementary Figure 12. **Enzyme activities of carbon-hydrolyzing enzymes (A, B), lignin-oxidizing enzyme (C), and enzymes hydrolyzing nitrogen (D) and phosphorus (E) compounds.** Significant differences ($p < 0.05$) from Kruskal–Wallis and Dunn's tests (Holm's *p* adjustment) indicated with lowercase letters (a, b). Error bars display standard error.

Supplementary Table 1. **List of publications included in the meta-analysis to quantify the SOC and N losses for *Kobresia pygmaea*'s core area.**

Authors	Year	Journal	Title	Pages
Ma Y, et al.	2002	Pratacultural Science	Study on rehabilitating and rebuilding technologies for degenerated alpine meadow in the Changjiang and Yellow river source region	1-5
Zhou H, et al.	2005	Acta Prataculturae Sinica	A study on correlations between vegetation degradation and soil degradation in the 'alpine meadow' of the Qinghai-Tibetan Plateau	31-40
Wang W, et al.	2006	Ecology and Environment	Effects of land degradation and rehabilitation on soil carbon and nitrogen content on alpine Kobresia meadow	362-366
Wang J, et al.	2007	Chinese Science Bulletin	Effects of swamp and alpine meadow degradation on CO_2 emission during growing season in Qinghai-Tibetan Plateau	1554-1560
Wang JF, et al.	2007	Chinese Science Bulletin	Influences of the degradation of swamp and alpine meadows on CO_2 emission during growing season on the Qinghai-Tibet Plateau	2565-2574
Wang W, et al.	2007	Journal of Plant Ecology (Chinese Version)	Effects of land degradation and rehabilitation on vegetation carbon and nitrogen content of alpine meadow in China	1073-1078
Zhao D & Xu Z	2007	Prataculture & Animal Husbandry	Research on the effect of degradation degree of subalpine meadow on soil nutrient in Laji Mountain Area	4-7
Li Y, et al.	2008	Chinese Journal of Grassland	Effect of typical alpine meadow degradation on soil enzyme and soil nutrient in source region of three rivers	51-58
Liu X, et al.	2008	Acta Prataculturae Sinica	Community structure and plant diversity of alpine meadow under different degrees of degradation in the Eastern Qilian Mountains	1-11
Wang C, et al.	2008	Chinese Journal of Applied and Environmental Biology	Changes in soil organic carbon and microbial biomass carbon at different degradation successional stages of alpine meadows in the headwater region of three rivers in China	225-230
Liu Y, et al.	2009	Journal of Agro-Environment Science	Vegetation decline and reduction of soil organic carbon stock in high-altitude meadow grasslands in the source area of Three Major Rivers of China	2559-2567
Wang CT, et al.	2009	Land Degradation & Development	Changes in plant diversity, biomass and soil C, in alpine meadows at different degradation stages in the headwater region of three rivers, China	187-198
Wang J, et al.	2009	China Environmental Science	Influence of degradation of the swamp and alpine meadows on CH_4 and CO_2 fluxes on the Qinghai-Tibetan Plateau	474-480
Xing X	2009	Journal of Anhui Agricultural Sciences	Different stages of degradation characteristics of alpine meadow in the Yellow River Source Area	10578-10580
Sheng L & Wang Y	2010	Chinese Qinghai Journal of Animal and Veterinary Sciences	Characteristics variation of soil nutrition in different degraded degree alpine meadow in Dawu Region of Guoluo Prefecture	4-6
Wang J, et al.	2010	Environmental Earth Sciences	The influence of degradation of the swamp and alpine meadows on CH_4 and CO_2 fluxes on the Qinghai-Tibetan Plateau	537-548
Cao L, et al.	2011	Pratacultural Science	Distribution of soil organic carbon and its relationship with soil physical and chemical properties on degraded alpine meadows	1411-1415

Yu J & Shi H	2011	Acta Agroculturae Boreali-occidentalis Sinica	Changes of microbes' population in the different degraded alpine meadows on the Qinghai-Tibetan Plateau	77-81
Luo Y, et al.	2012	Chinese Journal of Ecology	Soil stoichiometry characteristics of alpine meadow at its different degradation stages	254-260
Wang Y, et al.	2012	Environmental Earth Sciences	Correlation of alpine vegetation degradation and soil nutrient status of permafrost in the source regions of the Yangtze River, China	1215-1223
Yi XS, et al.	2012	Procedia Environmental Sciences	The impacts of grassland vegetation degradation on soil hydrological and ecological effects in the source region of the Yellow River-A case study in Junmuchang region of Maqin Country	967-981
Yu X, et al.	2012	Journal of Geo-Information Science	Spectral analysis of different degradation level alpine meadow in 'Three-River headwater' region	398-404
Zhang S, et al.	2012	Pratacultural Science	Soil nutrient characteristics of alpine meadow at different degradation degrees in Eastern Qilian Mountains	1028-1032
Zhang Z	2012	Heilongjiang Animal Science and Veterinary Medicine	Vegetation community structure and productivity of different degraded alpine meadow	83-85
Li Y, et al.	2013	Journal of Environmental Management	The effects of fencing on carbon stocks in the degraded alpine grasslands of the Qinghai-Tibetan Plateau	393-399
Wen L, et al.	2013	Plosone	Effect of degradation intensity on grassland ecosystem services in the alpine region of Qinghai-Tibetan Plateau, China	e58432
Wen L, et al.	2013	Plant and Soil	The impact of land degradation on the C pools in alpine grasslands of the Qinghai-Tibet Plateau	329-340
Zeng C, et al.	2013	Journal of Hydrology	Impact of alpine meadow degradation on soil hydraulic properties over the Qinghai-Tibetan Plateau	148-156
Li Y, et al.	2014	Geoderma	Soil carbon and nitrogen pools and their relationship to plant and soil dynamics of degraded and artificially restored grasslands of the Qinghai-Tibetan Plateau	178-184
Lu J, et al.	2014	Environmental Earth Sciences	The effect of desertification on carbon and nitrogen status in the northeastern margin of the Qinghai-Tibetan Plateau	807-815
Wang X, et al.	2014	Environmental Monitoring and Assessment	The effects of grassland degradation on plant diversity primary productivity, and soil fertility in the alpine region of Asian's headwaters	6903-6917
Wu GL, et al.	2014	Clean Soil Air Water	Above- and belowground response along degradation gradient in an alpine grassland of the Qinghai-Tibetan Plateau	319-323
Li J, et al.	2015	Ecological Research	Response of the plant community and soil water status to alpine Kobresia meadow degradation gradients on the Qinghai–Tibetan Plateau, China	589-596
Li Y, et al.	2015	Soil Biology &Biochemistry	Seasonal changes of CO_2, CH_4 and N_2O fluxes in different types of alpine grassland in the Qinghai-Tibetan Plateau of China	306-314
Lin L, et al.	2015	Solid Earth	Predicting parameters of degradation succession processes of Tibetan Kobresia grasslands	1237-1246
Liu Y, et al.	2015	Acta Agriculturae Boreali-occidentalis Sinica	Characteristics of soil organic carbon fractions in alpine meadow with different degradation	168-174

Liu Y, et al.	2015	Hubei Agricultural Sciences	Distribution characteristics of soil carbon on different degraded degree alpine meadow in the source area of Three Major Rivers in China	308-312
Shang ZH, et al.	2015	Plant Ecology & Diversity	Recruitment of seedlings versus ramets as affected by pasture degradation in alpine meadows and the implications for ecological restoration	547-557
Su X, et al.	2015	Journal of Mountain Sciences	Effects of grassland degradation and re-vegetation on carbon and nitrogen storage in the soils of the headwater area nature reserve on the Qinghai-Tibetan Plateau, China	582-591
Wu P, et al.	2015	Applied Soil Ecology	The response of soil macroinvertebrates to alpine meadow degradation in the Qinghai-Tibetan Plateau, China	60-67
Yu H, et al.	2015	Acta Agrestia Sinica	Distribution patterns of ratio of root to soil and soil physical chemical characteristics at the different degraded successional stages in an alpine meadow	1151-1160
Li Y, et al.	2016	Agriculture, Ecosystems & Environment	Changes of soil microbial community under different degraded gradients of alpine meadow	213-222
Liu SL, et al.	2016	Pratacultural Science	Effects of grazing season and degradation degree on the soil organic carbon in alpine meadow	11-18
Shang Z, et al.	2016	Ecological Engineering	Soil seed bank and its relation with above-ground vegetation along the degraded gradients of alpine meadow	268-277
Li W, et al.	2018	Ecological Engineering	Effect of degradation and rebuilding of artificial grasslands on soil respiration and carbon and nitrogen pools on an alpine meadow of the Qinghai-Tibetan Plateau	134-142
Lai ZM, et al.	2019	Pratacultural Science	Alpine meadows at different levels of degradation in the Beiluhe Basin of Tibetan Plateau Characteristics of soil respiration	952-959
Yang J, et al.	2020	Chinese Journal of Applied Ecology	Plant community and soil nutrient of alpine meadow in different degradation stages on the Tibetan Plateau, China	4067-4072
Yuan ZQ, et al.	2020	Journal of Soils and Sediments	Pasture degradation impact on soil carbon and nitrogen fractions of alpine meadow in a Tibetan permafrost region	2330-2342
Zhang ZH, et al.	2020	Journal of Arid Land	Degradation leads to dramatic decrease in topsoil but not subsoil root biomass in an alpine meadow on the Tibetan Plateau, China	806-818

Supplementary Table 2. **Soil and plant characteristics with successive degradation (own field study).** Values are means ± SE. Lowercase letters indicate significant differences between degradation stages ($p < 0.05$). Missing values with increasing degradation result from erosion removing the upper soil horizons.

	Degradation stage					
	**S0**	**S1**	**S2**	**S3**	**S4**	**S5**
**Horizontal crack (cm)**						
	-	4.0 (0.9)^{c}	6.6 (0.6)^{bc}	10.3 (1.9)^{ab}	17 (1.2)^{a}	-
**Vertical crack (cm)**						
	-	0.6 (0.1)^{e}	2.0 (0.3)^{d}	5.8 (0.3)^{c}	8.0 (0.4)^{b}	10.4 (0.8)^{a}
**Vegetation cover (%)**						
	95 (0.6)^{a}	92 (1.0)^{ab}	79 (3.5)^{b}	30 (3.0)^{c}	24 (2.9)^{c}	9 (0.9)^{d}
**Shoot biomass (g m^{-2})**						
	179 (11)^{ab}	210 (8)^{a}	70 (4)^{bc}	41 (5)^{c}	48 (11)^{cd}	22 (1)^{d}
**Root biomass in 0–25 cm (kg m^{-2})**						
	5.2 (0.12)^{a}	5.4 (0.06)^{a}	4.1 (0.33)^{b}	3.2 (0.42)^{b}	0.15 (0.02)^{c}	0.17 (0.01)^{c}
**Root density (mg cm^{-3})**						
**Ah1**	31 (2.8)^{a}	33 (2.5)^{a}				
**Ah2**	34 (1.7)^{ab}	32 (2.2)^{a}	28 (2.3)^{b}	28 (1.3)^{c}		
**Ah3**	23 (0.4)^{a}	22 (0.9)^{ab}	19 (1.4)^{bc}	16 (1.1)^{ab}	1.5 (0.2)^{d}	1.3 (0.1)^{cd}
**Ah4**	12 (1.1)^{a}	13 (1.1)^{ab}	12 (1.0)^{ab}	11 (0.8)^{ab}	0.6 (0.1)^{bc}	0.9 (0.1)^{c}
**Bulk density (mg cm^{-3})**						
**Ah1**	0.62 (0.01)^{a}	0.72 (0.14)^{a}				
**Ah2**	0.72 (0.07)^{a}	0.77 (0.09)^{a}	0.68 (0.01)^{a}	0.84 (0.00)^{a}		
**Ah3**	1.03 (0.06)^{a}	1.03 (0.06)^{a}	0.96 (0.01)^{ab}	0.82 (0.10)^{b}	0.98 (0.07)^{a}	1.24 (0.06)^{c}
**Ah4**	1.19 (0.02)^{ab}	1.06 (0.12)^{b}	1.13 (0.01)^{b}	1.24 (0.05)^{ab}	1.26 (0.07)^{ab}	1.37 (0.02)^{a}
**Bwg**	1.54 (0.02)^{a}	1.54 (0.04)^{a}	1.43 (0.01)^{a}	1.51 (0.08)^{a}	1.59 (0.06)^{a}	1.59 (0.03)^{a}
**SOC (g C kg^{-1})**						
**Ah1**	74 (4.6)^{a}	74 (6.0)^{a}				
**Ah2**	70 (0.7)^{a}	65 (5.0)^{ab}	53 (2.9)^{bc}	51 (4.6)^{c}		
**Ah3**	46 (2.1)^{a}	42 (2.2)^{a}	32 (3.4)^{b}	27 (4.6)^{b}	26 (2.3)^{b}	15 (1.4)^{c}
**Ah4**	25 (1.4)^{a}	19 (3.2)^{ab}	17 (2.4)^{ab}	13 (4.6)^{b}	19 (1.5)^{ab}	14 (1.4)^{b}
**Bwg**	4 (0.4)^{a}	4 (0.4)^{a}	5 (4.6)^{a}	5 (1.0)^{a}	5 (0.3)^{a}	4 (0.5)^{a}
**δ^{13}C of SOC (‰)**						
**Ah1**	-24.16 (0.12)^{a}	-24.28 (0.27)^{a}				
**Ah2**	-23.13 (0.11)^{a}	-23.69 (0.31)^{a}	-23.76 (0.34)^{a}	-24.02 (0.32)^{a}		
**Ah3**	-22.81 (0.08)^{a}	-23.30 (0.11)^{bc}	-23.56 (0.18)^{c}	-23.97 (0.18)^{d}	-24.30 (0.11)^{e}	-23.68 (0.10)^{cd}
**Ah4**	-22.90 (0.08)^{a}	-22.94 (0.09)^{a}	-23.21 (0.10)^{ab}	-23.58 (0.10)^{b}	-23.64 (0.08)^{b}	-23.19 (0.11)^{ab}
**Bwg**	-23.00 (0.10)^{a}	-22.84 (0.10)^{ab}	-22.91 (0.11)^{ab}	-23.01 (0.11)^{a}	-22.93 (0.11)^{a}	-22.62 (0.14)^{b}
**VSC of OC (mg g^{-1})**						
**Ah1**	11.1 (1.6)^{a}	11.8 (1.1)^{a}				
**Ah2**	9.8 (2.4)^{b}	9.9 (1.5)^{b}	9.8 (0.6)^{b}	18.3 (1.5)^{a}		
**Ah3**	3.7 (0.3)^{c}	6.4 (0.9)^{bc}	15.0 (0.9)^{abc}	24.3 (0.6)^{a}	19.4 (3.0)^{a}	21.4 (4.6)^{ab}
**Ah4**	6.5 (0.8)^{c}	8.3 (1.3)^{bc}	14.0 (2.3)^{abc}	15.1 (5.4)^{abc}	20.8 (3.5)^{a}	18.1 (4.8)^{ab}
**Bwg**	14.1 (1.6)^{a}	15.5 (1.5)^{a}	12.9 (2.4)^{a}	9.9 (2.1)^{a}	10.1 (3.9)^{a}	17.9 (3.9)^{a}

SOC, soil organic carbon; VSC, vanillyl, syringyl and cinnamyl units.

Supplementary Table 3. **Important microbial taxonomic groups described for the *Kobresia* ecosystem.**

Taxonomic group	Function	Literature
Actinobacteria	Degrading low-molecular-mass compounds	Šnajdr et al. (a)
Agaricomycetes	Lignin degrader	Ahmad et al. (b)
Ascomycota	Litter degrader	Rajala et al. (c)
Glomeromycota	AMF/VAM	Brundrett (d)
Inocybaceae	ECM partner of *Kobresia sp.*	Gao and Yang (e)
Nitrosomonadaceae	Nitrifier	Jetten et al. (f)
Nitrospirales	Nitrifier	Jetten et al. (f)
Pseudomonadales	Denitrifier, lignin degrader	Ahmad et al. (b)
Rhizobiales	Lignin degrader	Nacke et al. (g)
Thelephoraceae	ECM partner of *Kobresia* sp.	Gao and Yang (e)

a. Šnajdr, J. et al. Transformation of *Quercus petraea* litter: Successive changes in litter chemistry are reflected in differential enzyme activity and changes in the microbial community composition. *FEMS Microbiol. Ecol.* **75**, 291–303 (2011).

b. Ahmad, M. et al. Development of novel assays for lignin degradation: Comparative analysis of bacterial and fungal lignin degraders. *Mol. Biosyst.* **6**, 815–821 (2010).

c. Rajala, T., Peltoniemi, M., Pennanen, T. & Mäkipää, R. Fungal community dynamics in relation to substrate quality of decaying Norway spruce (*Picea abies* [L.] Karst.) logs in boreal forests. *FEMS Microbiol. Ecol.* **81**, 494–505 (2012).

d. Brundrett, M. Diversity and classification of mycorrhizal associations. *Biol. Rev.* **79**, 473–495 (2004).

e. Gao, Q. & Yang, Z. L. Ectomycorrhizal fungi associated with two species of *Kobresia* in an alpine meadow in the eastern Himalaya. *Mycorrhiza* **20**, 281–287 (2010).

f. Jetten, M. S. M. et al. Improved nitrogen removal by application of new nitrogen-cycle bacteria. *Rev. Environ. Sci. Biotechnol.* **1**, 51–63 (2002).

g. Nacke, H., Fischer, C., Thürmer, A., Meinicke, P. & Daniel, R. Land use type significantly affects microbial gene transcription in soil. *Microb. Ecol.* **67**, 919–930 (2014).
