# Supplementary material for: Microbial functional changes mark irreversible course of Tibetan grassland degradation
Source: Nat Commun. 2022 May 13;13:2681. doi: 10.1038/s41467-022-30047-7 (PMC9106683; doi:10.1038/s41467-022-30047-7)
Supplement: Supplementary file 2 — Reporting Summary [file 41467_2022_30047_MOESM2_ESM.pdf]

Corresponding author(s): Sandra Spielvogel

Last updated by author(s): Mar 10, 2022

Reporting Summary

Nature Portfolio wishes to improve the reproducibility of the work that we publish. This form provides structure for consistency and transparency in reporting. For further information on Nature Portfolio policies, see our Editorial Policies and the Editorial Policy Checklist.

Statistics

For all statistical analyses, confirm that the following items are present in the figure legend, table legend, main text, or Methods section.

n/a	Confirmed	
☐	☒	The exact sample size (n) for each experimental group/condition, given as a discrete number and unit of measurement
☐	☒	A statement on whether measurements were taken from distinct samples or whether the same sample was measured repeatedly
☐	☒	The statistical test(s) used AND whether they are one- or two-sided<br>*Only common tests should be described solely by name; describe more complex techniques in the Methods section.*
☐	☒	A description of all covariates tested
☐	☒	A description of any assumptions or corrections, such as tests of normality and adjustment for multiple comparisons
☐	☒	A full description of the statistical parameters including central tendency (e.g. means) or other basic estimates (e.g. regression coefficient) AND variation (e.g. standard deviation) or associated estimates of uncertainty (e.g. confidence intervals)
☐	☒	For null hypothesis testing, the test statistic (e.g. F, t, r) with confidence intervals, effect sizes, degrees of freedom and P value noted<br>*Give P values as exact values whenever suitable.*
☒	☐	For Bayesian analysis, information on the choice of priors and Markov chain Monte Carlo settings
☒	☐	For hierarchical and complex designs, identification of the appropriate level for tests and full reporting of outcomes
☐	☒	Estimates of effect sizes (e.g. Cohen's d, Pearson's r), indicating how they were calculated

Our web collection on statistics for biologists contains articles on many of the points above.

Software and code

Policy information about availability of computer code

Data collection	not applicable
Data analysis	Statistical analyses were performed using PASW Statistics (IBM SPSS Statistics) and R software (Version 3.6.1).

For manuscripts utilizing custom algorithms or software that are central to the research but not yet described in published literature, software must be made available to editors and reviewers. We strongly encourage code deposition in a community repository (e.g. GitHub). See the Nature Portfolio guidelines for submitting code & software for further information.

Data

Policy information about availability of data

All manuscripts must include a data availability statement. This statement should provide the following information, where applicable:

- Accession codes, unique identifiers, or web links for publicly available datasets
- A description of any restrictions on data availability
- For clinical datasets or third party data, please ensure that the statement adheres to our policy

The data generated in this study have been deposited in the PANGAEA Open Access library under accession code https://doi.pangaea.de/10.1594/PANGAEA.918249. The 16S rRNA and ITS gene paired-end raw reads for the bacterial and fungal community analyses have been deposited in the National Center for Biotechnology In-formation (NCBI) Sequence Read Archive (SRA) under the accession code PRJNA626504; BioProject: Tibetan plateau microbiome, https://www.ncbi.nlm.nih.gov/bioproject/PRJNA626504.

Field-specific reporting

Please select the one below that is the best fit for your research. If you are not sure, read the appropriate sections before making your selection.

☐ Life sciences ☐ Behavioural & social sciences ☒ Ecological, evolutionary & environmental sciences

For a reference copy of the document with all sections, see nature.com/documents/nr-reporting-summary-flat.pdf

Ecological, evolutionary & environmental sciences study design

All studies must disclose on these points even when the disclosure is negative.

Study description

Literature study
Literature considering the effect of pasture degradation on SOC, N and clay content as well as bulk density (BD) was assembled. Search terms were "degradation gradient", "degradation stages", "alpine meadow", "Tibetan Plateau", "soil", "soil organic carbon" and "soil organic matter" in different combinations. The degradation stages in the literature studies were regrouped into the six successive stages according to the respective degradation descriptions. In total, we compiled the results of 49 publications published between 2002 and 2020.
Experimental design of the field study
Large areas in the study region are impacted by grassland degradation. In total, 45% of the surface area of the Kobresia pasture ecosystem on the TP is already degraded. The experiment was designed to differentiate and quantify SOC losses by erosion vs. net decomposition, and to identify underlying shifts in microbial community composition and link these to changes in key microbial functions in the soil C cycle.

Research sample

Literature study
Literature considering the effect of pasture degradation on SOC, N and clay content as well as bulk density (BD) was assembled by searching (i) Web of Science V.5.22.1, (ii) ScienceDirect (Elsevier B.V.) (iii) Google Scholar, and (iv) the China Knowledge Resource Integrated Database (CNKI). Search terms were "degradation gradient", "degradation stages", "alpine meadow", "Tibetan Plateau", "soil", "soil organic carbon" and "soil organic matter" in different combinations. The criteria for including a study in the analysis were: (i) a clear and comprehensible classification of degradation stages was presented, (ii) data on SOC, N, and/or BD were reported, (iii) a non-degraded pasture site was included as reference to enable an effect size analysis and the calculation of SOC and N losses, (iv) sampling depths and study location were clearly presented. (v) Studies were only considered that took samples in 10 cm depth intervals, to maintain comparability to the analyses from our own study site.
Experimental field study
We categorized the range of Kobresia root mat degradation from non-degraded to bare soils into six successive degradation stages (S0 to S5). Stage S0 represented non-degraded root mats, while stages S1 to S4 represented increasing degrees of surface cracks, and bare soil patches without root mats defined stage S5. All six degradation stages were selected within an area of about 4 ha to ensure equal environmental conditions and each stage was sampled in four field replicates. However, the studied degradation patterns are common for the entire Kobresia ecosystem.

Sampling strategy

Sample sizes were selected according to standards in ecological/biogeochemical studies allowing for a robust statistical evaluation of treatment-based datasets considering natural variablity to be expected in the respective study area. Sample depth was maximized (i.e. sampling until bedrock was performed).
Soil sampling was conducted using soil pits (30 cm length × 30 cm width × 40 cm depth). Horizons were classified and then soil and roots were sampled for each horizon directly below the cracks. Bulk density and root biomass were determined in undisturbed soil samples, using soil cores (10 cm height and 10 cm diameter). Living roots were separated from dead roots and root debris by their bright color and soft texture using tweezers under magnification, and the roots were subsequently washed with distilled water to remove remaining soil. Because over 95% of the roots occurred in the upper 25 cm (5), we did not sample for root biomass below this depth. Additional soil samples were taken from each horizon for further analysis. Microbial community and functional characterization were performed on samples from the same pits but with a fixed depth classification (0–5 cm, 5–15 cm, 15–35 cm) to reduce the number of samples.

Data collection

Literature study
Data were collected and analyzed by Shibin Liu as described above.
Experimental field study:
Soil samples for basic soil parameter analysis (C, N, P content, pH, delta13C, delta15N and lignin phenol analysis) were taken by Per Schleuss and Sandra Spielvogel as described above. Soil samples for microbiological analysis (DNA extraction and sequencing) have been taken by Sandra Spielvogel and analyzed by Jianchu Xu. Samples for enzyme analysis were sampled by Sandra Spielvogel and directly analyzed on site.

Timing and spatial scale

Literature study
Studies comprising site from those areas of the Tibetan Plateau which were covered with Koberesia pygemea pastures were considered. We only included those studies that took samples in 10 cm depth intervals, to maintain comparability to the analyses from our own study site.
Experimental field site description
The field study was conducted near Nagqu (Tibet, China) in late summer 2013 and 2015. The study site of about 4 ha (NW: 31.274748°N, 92.108963°E; NE: 31.274995°N, 92.111482°E; SW: 31.273488°N, 92.108906°E; SE: 31.273421°N, 92.112025°E) was located on gentle slopes (2–5%) at 4,484 m a.s.l. in the core area of the Kobresia pygmaea ecosystem.

Data exclusions

No data were excluded from the field study. Data selection criteria of the literature study are described above.

Reproducibility	Standardized, long-term in-use methods were applied to produce the here reported data. Basic soil characteristics and enzyme analysis were performed at least in duplicate often triplicates reaching high reproducabilty. Mean values were used for further data analysis.
Randomization	Full randomization of all samples was ensured for all reported analytical methods.
Blinding	Lab codes containing no treatment identifier were given to all samples before analysis.

Did the study involve field work? ☒ Yes ☐ No

Field work, collection and transport

Field conditions	Sampling occured during the dry season of the Tibetan Pleatau. Temperatures ranged between 10-15 degrees.
Location	The field study was conducted near Nagqu (Tibet, China) in late summer 2013 and 2015. The study site of about 4 ha (NW: 31.274748°N, 92.108963°E; NE: 31.274995°N, 92.111482°E; SW: 31.273488°N, 92.108906°E; SE: 31.273421°N, 92.112025°E) was located at 4,484 m a.s.l. in the core area of the Kobresia pygmaea ecosystem.
Access & import/export	All samples have been taken from pasture ecosystems. None of the sampling locations was a protected habitat area. Work permits were provided with the issued working visa for the Tibetan Plateau issued by the Chinese Embassy of Frankfurt (a. M.). Microbiological analyses were carried out within the country (China). Export declaration for geological samples were issued by the Chinese Academy of Sciences (Prof. Xingliang Xu).
Disturbance	No additional disturbance occured as the study took place at already highly disturbed sites.

Reporting for specific materials, systems and methods

We require information from authors about some types of materials, experimental systems and methods used in many studies. Here, indicate whether each material, system or method listed is relevant to your study. If you are not sure if a list item applies to your research, read the appropriate section before selecting a response.

Materials & experimental systems

n/a	Involved in the study	
☒	☐	Antibodies
☒	☐	Eukaryotic cell lines
☒	☐	Palaeontology and archaeology
☒	☐	Animals and other organisms
☒	☐	Human research participants
☒	☐	Clinical data
☒	☐	Dual use research of concern

Methods

n/a	Involved in the study	
☒	☐	ChIP-seq
☒	☐	Flow cytometry
☒	☐	MRI-based neuroimaging
